# Examining a Telemedicine-Based Virtual Reality Clinic in Treating Adults With Specific Phobia: Protocol for a Feasibility Randomized Controlled Efficacy Trial

**DOI:** 10.2196/65770

**Published:** 2025-04-10

**Authors:** Kaitlyn R Schuler, Triton Ong, Brandon M Welch, Jason G Craggs, Brian E Bunnell

**Affiliations:** 1 Department of Psychology University of North Carolina at Wilmington Wilmington, NC United States; 2 Doxy.me Research Rochester, NY United States; 3 Doxy.me Charleston, SC United States; 4 Department of Public Health Sciences Medical University of South Carolina Charleston, SC United States; 5 Department of Psychiatry and Behavioral Neurosciences Morsani College of Medicine University of South Florida Tampa, FL United States

**Keywords:** virtual reality, exposure therapy, phobias, telemedicine, telemental health, tele-VR, immersive simulations

## Abstract

**Background:**

Virtual reality (VR) has strong potential to enhance the effectiveness of telemental health care (TMH) by providing accessible, personalized treatment from home. While there is ample research supporting VR for in-person treatment, there is only preliminary data on the efficacy of telemedicine-based VR. Furthermore, the majority of VR apps used in therapy are not designed for mental health care. VR has the potential to enhance TMH through innovative technology solutions designed specifically for the enhancement of remotely delivered evidence-based practices. This feasibility randomized controlled efficacy trial aims to fill both of these gaps by piloting a novel telemedicine-based VR app (Doxy.me VR) equipped with animal phobia exposure stimuli.

**Objective:**

This is a feasibility randomized controlled efficacy trial comparing exposure therapy via a telemedicine-based VR clinic versus standard TMH with adults with an intense fear of dogs, snakes, or spiders. The primary objective is to assess the feasibility of a fully powered trial. The secondary objective is to conduct a preliminary examination of clinical outcomes (eg, specific phobia symptoms).

**Methods:**

This single-site trial will enroll a minimum of 30 and a maximum of 60 adults with self-reported fear of dogs, snakes, or spiders. Potential participants will be recruited through clinical trial and research recruitment websites and posting flyers. All self-report assessments and homework will be partially automated using REDCap (Research Electronic Data Capture; Vanderbilt University) forms and surveys, but the baseline assessment of phobia symptoms and exposure intervention will be administered by the study therapist.

**Results:**

The feasibility of the proposed trial methodology will be assessed using enrollment, retention, assessment completion, and treatment protocol fidelity benchmarks. Between-group differences in specific phobia, anxiety, and depression symptoms while covarying for pretreatment scores, will be conducted using repeated measures ANOVA along with differences in therapeutic alliance and presence. Data obtained from these analyses will inform power analyses for a fully powered efficacy trial. In total, 54 participants were randomized between October 25, 2023, and July 26, 2024 (Doxy.me VR n=28 and TMH n=26). Data analysis will be completed and submitted by the end of the second quarter of 2025.

**Conclusions:**

This feasibility randomized controlled trial comparing Doxy.me VR versus TMH aims to enhance the delivery of evidence-based treatments via telemedicine and reduce barriers to remotely delivered exposure therapy. This feasibility trial will be followed by a fully powered efficacy trial on telemedicine-based VR for animal phobias.

**Trial Registration:**

ClinicalTrials.gov NCT06302868; https://clinicaltrials.gov/study/NCT06302868

**International Registered Report Identifier (IRRID):**

DERR1-10.2196/65770

## Introduction

### Background

Telemental health care (TMH) has revolutionized mental health services by providing accessible, personalized treatment from the comfort and privacy of home [1,2]. TMH is equally to more effective than in-person care, with patients generally reporting higher satisfaction and reduced costs [3-11]. Furthermore, TMH alleviates sociocultural (eg, stigma) and geographic (eg, transportation) barriers to mental health care [12-14]. TMH increased from 41.8% to 62.8% of all telehealth services during 2022, surpassing all other forms of digital clinical care [15]. This trend suggests that clients view TMH as an appropriate substitute for in-person care. As TMH services continue to evolve, there will be a growing need for innovative solutions to enhance the effectiveness of remote care [1,16,17].

Virtual reality (VR) is a promising avenue for advancing TMH. By leveraging immersive simulations, VR can recreate stressors—both physical and psychological—in a safe, controlled environment, which can enhance the effectiveness of remotely delivered evidence-based treatments [18-21]. VR improves treatment compliance and patient retention by offering engaging content for in- and between-session mental health exercises [22]. There is abundant support for on-site VR mental health therapy [23,24]; however, evidence for telemedicine-based VR mental health is limited to a few studies of preliminary efficacy [25-27]. VR has strong potential to drive innovations in TMH [28,29].

The customizable simulations of VR can create new opportunities to personalize TMH. For example, exposure therapy is a gold standard treatment for phobias and other anxiety-related mental health disorders that is efficient and produces durable effects [30,31]. By exposing an individual to fear-related stimuli (eg, a dog) in a controlled setting (eg, the therapist’s office), anxiety reduces, and the fear response diminishes over prolonged and repeated exposure (eg, the individual is no longer fearful around dogs). One approach to conducting gradual exposure includes using multimedia stimuli (eg, screen-shared photos or videos), but it can be challenging to standardize exposure formats and assess client affect over conventional telemedicine [32,33]. Another approach is exposing patients to their fears using VR. VR-based exposure therapy (VRET) is a safe, accessible, and engaging alternative to in vivo exposure therapy that can be less stressful and more conducive to treatment success [34-37]. However, most VRET research to date has been conducted in-person, requiring that patients travel to and complete therapy in a clinician’s office [38]. Delivering VRET over telemedicine may provide patients with highly engaging and immersive treatment with greater accessibility [39]. Telemedicine-based VR may also address lingering barriers to TMH by expanding the experience of therapeutic alliance and presence. Therapeutic alliance, or working alliance, refers to the extent to which a patient and therapist experience a collaborative and trusting relationship [40,41] and is among the strongest predictors of outcomes in mental health care [42,43]. Presence, also referred to as telepresence, is the extent to which places, activities, and other individuals feel real in a digital environment (eg, VR and videoconferencing) [44].

To explore the potential of telemedicine-based VR therapy, we conducted in-depth interviews with 18 practicing TMH therapists who had delivered exposure therapy via conventional telemedicine [45]. Therapists wanted VR features to build rapport with their patients and the ability to customize VR components for individual patient needs. Therapists needed clinical evidence supporting tele-VR, low costs to adopt VR into their TMH practice, and information on possible side effects for patients. We then surveyed 176 practicing TMH therapists about their perspectives on telemedicine-based VR. We asked therapists to rate the relative importance of tele-VR simulations, features, and implementation factors. Therapists strongly favored tele-VR simulations related to social anxiety, flying, driving, and animals. Therapists rated between-session VR exercises and immersive cooperative activities as some of their most valued tele-VR features. Therapists’ highest-rated factors for implementing telemedicine-based VR were HIPAA (Health Insurance Portability and Accountability Act)-compliant, followed by affordability, insurance coverage, and accessibility [46].

**Figure 1:**
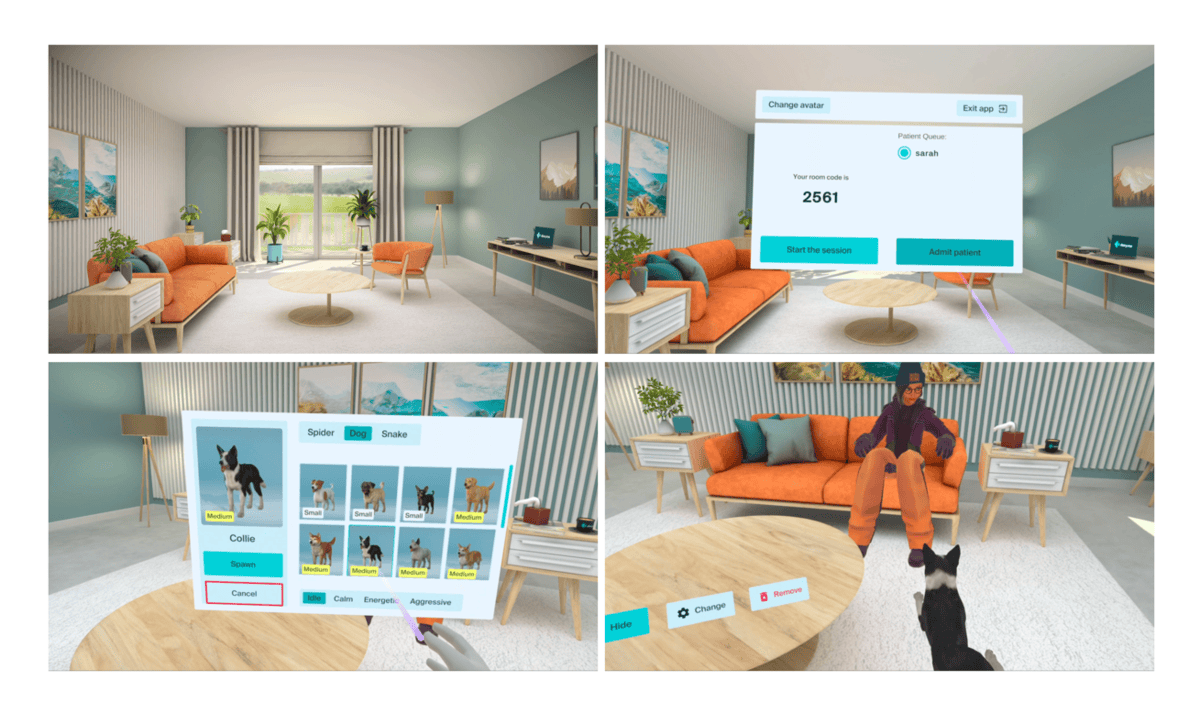
Screenshots from Doxy.me VR. Virtual reality clinic space (top left); client admission menu (top right); phobia stimulus menu (bottom left); and therapist and client interacting with phobic stimulus in virtual reality (lower right).

Using insights obtained from these studies, we developed Doxy.me VR—an innovative app developed specifically to facilitate immersive TMH. We developed Doxy.me VR with the frequent and direct involvement of key stakeholders. Doxy.me VR is designed for a therapist to meet with a client remotely in a private, comfortable VR clinic that looks and feels like a therapist’s office (refer to Figure 1 for screenshots of the clinic). To join the VR session, the therapist provides their unique, persistent 4-digit room code to their client, who enters the code to check in. Once the therapist admits the client, they can interact by speaking and gesturing with each other in immersive VR. Therapists can use a menu to spawn animals such as dogs, snakes, and spiders for use in treating specific phobias. Multiple exemplars are available for each type of animal in varying sizes. For example, there are small dogs (eg, Chihuahua, Corgi, and Jack Russell Terrier), medium dogs (eg, Golden Retriever, Shiba Inu, and Pit Bull), and large dogs (eg, German Shepherd, Doberman, and Husky). Each animal is animated with 4 behavior states, that is, idle (ie, no movement), calm (ie, small, slow movements in a relaxed posture), active (ie, fast, frequent movements in a playful posture), and aggressive (ie, fast, attacking movements in a defensive posture). Each behavior state also includes corresponding audio such as a calm dog breathing lightly, an active dog panting and barking playfully, and an aggressive dog snarling and barking loudly. Therapists can select, rotate, and move these animals around the room before making them visible to clients. Once therapists make the animal visible, they can continue to move and rotate the animal or remove the animal entirely. Clients cannot directly manipulate objects; however, clients gain access to all the control features while engaging with Doxy.me VR’s homework mode, which is used for between-session practice.

### This Study

We present the protocol for a feasibility randomized controlled efficacy trial comparing exposure therapy delivered via Doxy.me VR versus standard TMH to adults with an intense fear of dogs, snakes, or spiders. The primary aim of this study is to assess the feasibility of and refine our study methodology in preparation for a large, fully powered randomized controlled efficacy trial. The secondary aim is to conduct a preliminary examination of clinical outcomes, including between-group differences in specific phobia symptoms, therapeutic alliance, and presence.

## Methods

### Enrollment and Randomization

This study will use a feasibility randomized controlled efficacy trial design. This single-site trial will enroll a maximum of 60 adults with self-reported fear of dogs, snakes, or spiders with the goal of completing treatment with 30 adults. Participants will be randomly assigned using the REDCap (Research Electronic Data Capture; Vanderbilt University) [47-49]. Randomization Module on a 1:1 allocation ratio to receive 12 weekly sessions of exposure therapy over the course of three months delivered via standard TMH (n=15) versus Doxy.me VR (n=15). Participants randomized to the Doxy.me group will be provided with Meta Quest 2 VR headsets preloaded with the Doxy.me VR app, to be returned to the study team following completion of the study. All therapy sessions will be conducted via remote videoconferencing, with the Doxy.me VR group transitioning to the Doxy.me VR clinic for the exposure exercise portion of the session, and the standard TMH group using multimedia (ie, photos and videos) shared by the study therapist during video calls. Participants randomized to Doxy.me VR received a Meta Quest 2 headset via mail with the VR clinic already downloaded and were provided a clinic code by their therapist at the beginning of each session.

### Study Intervention

This study will follow the treatment protocol developed specifically for VRET by Bouchard et al [50], Côté and Bouchard [51], Michaliszyn et al [52], and St-Jacques et al [53]. Following the baseline assessment session, treatment consists of 12, ≤60-minute therapy sessions, including 1 psychoeducation and treatment planning session (session 1), 10 exposure sessions (sessions 2-11), and 1 relapse prevention session (session 12). During the first therapy session, patients learn about the principles of cognitive behavioral therapy, the etiology of anxiety and specific phobias, and procedures for exposure. They then complete a fear hierarchy (ie, a list of situations ranked from least to most anxiety-provoking) with therapist guidance. Fear hierarchies include a text-based list of various VR situations or multimedia situations (ie, static and active states of different types of feared animals) and “in vivo” or live situations (eg, petting a dog and touching a snake at a pet store). Therapy sessions 2-11 begin with a review of homework assigned during the previous week’s session, followed by an approximately 35-minute virtual exposure session. Patients report their anxiety level using a 1- to 100-point scale at a 5-minute interval throughout the exposure and remain in the exposure until their self-reported anxiety reaches a 50% reduction from their peak anxiety rating from baseline following the introduction of the stimulus or 35 minutes elapses. That is, participants will spend a maximum of 35 minutes in the exposure and if they have reached a 50% reduction or greater from their peak anxiety rating before the 35 minutes is over, they will move on to the next exposure. Following the exposure, the therapist and patient process thoughts and feelings related to the exposure, reframe appraisals of their feared stimuli through cognitive restructuring, and practice box breathing. The Doxy.me VR group will conduct their in-session exposures in the VR clinic and the TMH group will conduct their in-session exposures using multimedia on the Doxy.me platform by screen-sharing videos and photos. Once patients have progressed through all VR and multimedia situations in their hierarchy and are ready to complete their first in vivo exposure between therapy sessions for homework, weekly therapy sessions will be devoted to processing and planning between-session in vivo exposures.

### Homework

All therapy sessions will include the assignment of homework, including reading an informational handout after the psychoeducation and treatment planning session (session 1) and exposure therapy assignments after therapy sessions 2-11. Daily exposure-based homework assignments will require participants to practice exposure exercises similar to those completed during that week’s therapy session (ie, using VR or multimedia stimuli) for a minimum of 30 minutes. When the patient states that they are ready, these will include in vivo exposure exercises planned during the therapy session. Participants will receive text or email reminders with links to REDCap surveys with instructions for the homework assignment and the situation to be used in the exposure exercise. The survey will also ask participants to report the date and time of the exposure, their baseline anxiety rating, their peak anxiety level during the exposure, and their anxiety level following completion of the exposure exercise.

### Recruitment Strategy

Potential participants will be recruited through Clinical Connection [54], Research Match (retrieved from researchmatch website [55]), Facebook (Meta [56]) advertisements, and flyers distributed on the University of South Florida campus and off-campus community centers. Potential participants will be provided with a URL or QR code directing them to an online prebaseline screening questionnaire administered via REDCap. Those meeting preliminary eligibility criteria will then schedule an initial consent and baseline assessment visit via Microsoft Bookings with text or email reminders 24 hours and 1 hour before the scheduled visit time.

### Eligibility Criteria

Eligible participants will (1) be ≥18 years old; (2) have a self-reported fear of dogs, snakes, or spiders; (3) have subthreshold or present specific phobia symptoms as determined by the study therapist via administration of the Diagnostic Assessment Research Tool (DART) Specific Phobia Module [57]; (4) have access to the internet and a computer or smartphone with videoconferencing capabilities; and (5) plan to reside in the state of Florida for the duration of the study.

An individual who endorses any of the following criteria will be excluded from participation in the study: (1) participation in ongoing mental health therapy from a nonstudy therapist; (2) changes to psychotropic medication use within 6 weeks preceding enrollment in the trial; (3) active suicidal or homicidal intent or plan as determined by the study therapist via the DART Risk Assessment Module [57]; (4) active auditory, visual, or tactile hallucinations via the DART Psychosis Module screening question; or (5) a diagnosis of photosensitive epilepsy by a medical doctor or a history of experiencing seizures caused by photosensitivity.

### Criteria for Withdrawing Participants

Anticipated circumstances under which participants will be withdrawn without their consent include (1) new participation in mental health therapy from a nonstudy therapist; (2) changes to psychotropic medication use; (3) active suicidal or homicidal intent or plan; (4) onset of auditory, visual, or tactile hallucinations; (5) onset of photosensitive epilepsy or seizures; and (6) moving out of the State of Florida during the study period.

If participants completely withdraw or are administratively withdrawn from the study, staff will attempt to provide them with a referral to a therapist in their area. If participants partially withdraw from the research, study staff will attempt to administer mid- and posttreatment assessments. Participants will be given the option to completely withdraw from the study, including withdrawing previously collected data.

### Criteria for Wait-Listing Participants

If potential participants do not meet eligibility criteria due to participating in ongoing mental health therapy from a nonstudy therapist or recent changes to psychotropic medication use within 6 weeks preceding enrollment in the trial, they will be presented with a message on the prescreening survey inviting them to revisit the prescreening questionnaire at a later date if and when those conditions no longer apply.

### Consent and Baseline Assessments

The consent and baseline assessment visit will be conducted via videoconferencing platform and will not be audio or video-recorded. During this visit, study staff will (1) confirm the potential participant’s responses on the online prescreening questionnaire, (2) provide detailed information about the study and obtain informed consent, (3) assist participants in completing baseline questionnaires via REDCap survey, (4) administer the Specific Phobia and Risk Assessment modules of the DART, and (5) make a final determination on the participant’s eligibility. Eligible participants will then be randomized using the REDCap Randomization Module and scheduled for their first treatment visit. Neither participants nor study staff will be blinded to the comparator of interest or their group assignment.

### Assessment Strategy and Measures

#### Overview

All assessments were chosen considering several factors including (1) sound psychometric properties, (2) ease of administration, (3) past use in similar clinical trials, and (4) brevity. All assessments will be facilitated remotely by the study therapist and will occur at baseline, each session, midtreatment (ie, after completion of 6 therapy sessions, up to 6 weeks post baseline), and 12 weeks post baseline. Self-report questionnaires will be completed by patients via REDCap survey with the study therapist present to answer any questions. Structured diagnostic interviews will be administered by study therapists and recorded in REDCap.

#### Specific Phobia Symptoms and Severity

The DART [57] is a modular semistructured interview corresponding with the *DSM-5* (*Diagnostic and Statistical Manual of Mental Disorders* [Fifth Edition]) [58]. Study therapists will administer the DART Specific Phobia Module, which provides designations of absent, subthreshold, and present. While the DART is a new tool, initial validation indicates excellent construct validity, discriminant validity, and convergent validity across modules [57,59].

The Severity Measure for Specific Phobias (SMSP) for adults [60] is a 10-item self-report questionnaire assessing the severity of specific phobia symptoms as they relate to the respondent’s feared stimulus. Total scores range from 0 to 4 with higher scores indicating higher phobia severity. The SMSP has excellent internal consistency, criterion, and discriminant validity [61].

#### Other Mental Health Symptoms

##### Suicide and Homicide Risk

The DART-Risk Assessment Module [57] will be administered by study therapists to assess risk for suicidal and homicidal ideation, intention, and plans and guides the formation of safety plans where warranted.

##### Generalized Anxiety

The Generalized Anxiety Disorder-7 Scale (GAD-7) [62] is a 7-item self-report measure of anxiety symptom severity. Scores range from 0 to 15 with higher scores indicating higher anxiety severity. The GAD-7 is widely used and has demonstrated excellent internal consistency reliability and good convergent validity [63].

##### Depression

The Patient Health Questionnaire-8 (PHQ-8) [64] is an 8-item self-report measure of depression symptom severity. Scores range from 0 to 27 with higher scores indicating higher depression severity. The PHQ-8 has demonstrated excellent internal consistency reliability, good convergent validity, and specificity for depression diagnosis [65].

#### Treatment-Related Factors

##### Working Alliance

The Working Alliance Inventory [66] is a 10-item client-rated measure of therapeutic alliance. Total scores range from 1 to 5 with higher scores indicating a better therapeutic alliance. The WAI-SR has demonstrated excellent internal consistency, reliability, and good convergent validity [67].

##### Presence

The Single Item Presence Questionnaire (SIPQ) [68] is a 1-item self-reported measure of telepresence. Respondents are asked, “To what extent did you feel present in the environment, as if you were really there?” and provide ratings on a scale of 0 “not at all present” to 10 “totally present.” The SIPQ has demonstrated good to excellent content, face validity, test-retest, convergent and divergent validity, and sensitivity [68].

##### Cybersickness

The Cybersickness in Virtual Reality Questionnaire (CSQ-VR) [69] is an 8-item measure of nausea, vestibular, and oculomotor cybersickness experienced in VR. Scores range from 6 to 27 with higher scores indicating higher levels of cybersickness. The CSQ-VR has demonstrated excellent internal consistency and convergent validity [70].

##### Client Satisfaction With Treatment

The Client Satisfaction Questionnaire-8 (CSQ-8) [71] is an 8-item client-rated measure satisfaction with treatment. Scores range from 8 to 32 with higher scores indicating higher satisfaction. The CSQ-8 has demonstrated good structural validity and internal reliability and is correlated with clinical outcomes and posttreatment functioning [72].

##### System Usability

The System Usability Scale (SUS) [73] is a 10-item self-report measure of the usability of a particular software system, platform, or app. Scores range from 0 to 100 with higher scores indicating better usability. The SUS has demonstrated good internal consistency reliability and is a useful tool for comparing system usability between 2 and more platforms.

### Fidelity to the Treatment Protocol

All treatment sessions will be recorded and 20% (120/600) will be rated by the supervisor (principal investigator) using a treatment fidelity checklist based on the treatment manual. Refer to Table 1 for a summary of study assessments.

**Table 1:** Study assessments.

Domain	Measure	Time point				
		Pre	Baseline	Weekly	Mid	Post
Demographics	Demographics Questionnaire		✓			
Specific phobia diagnosis	Diagnostic Assessment Research Tool–Specific Phobia Module (DART-SP)		✓			✓
Specific phobia severity	Severity Measure for Specific Phobia-Adult (SMSP-A)	✓	✓		✓	✓
Risk	Diagnostic Assessment Research Tool–Risk Assessment (DART-RA)		✓			
Anxiety severity	General Anxiety Disorder-7 (GAD-7)		✓		✓	✓
Depression severity	Patient Health Questionnaire-9 (PHQ-9)		✓		✓	✓
Therapeutic alliance	Working Alliance Inventory- Short Revised (WAI-SR)				✓	✓
Presence	Single Item Presence Questionnaire (SIPQ)			✓		
Cybersickness	Cybersickness in Virtual Reality Questionnaire (CSQ-VR)			✓		
Treatment satisfaction	Client Satisfaction Questionnaire (CSQ-8)				✓	✓
Usability	System Usability Scale (SUS)				✓	✓

### Partial Automation of Trial Data Collection Using REDCap

All feasibility trial data will be collected via 2 REDCap projects. The first REDCap project will include a survey to collect responses to the online prescreening questionnaire, which will include questions about basic eligibility criteria, the SMSP, and contact information. If potential participants meet the initial study eligibility criteria of the REDCap survey, they will be directed to a Microsoft Bookings page to schedule their baseline assessment, with automated reminders about the appointment 24 and 1 hours before the appointment.

**Figure 2:**
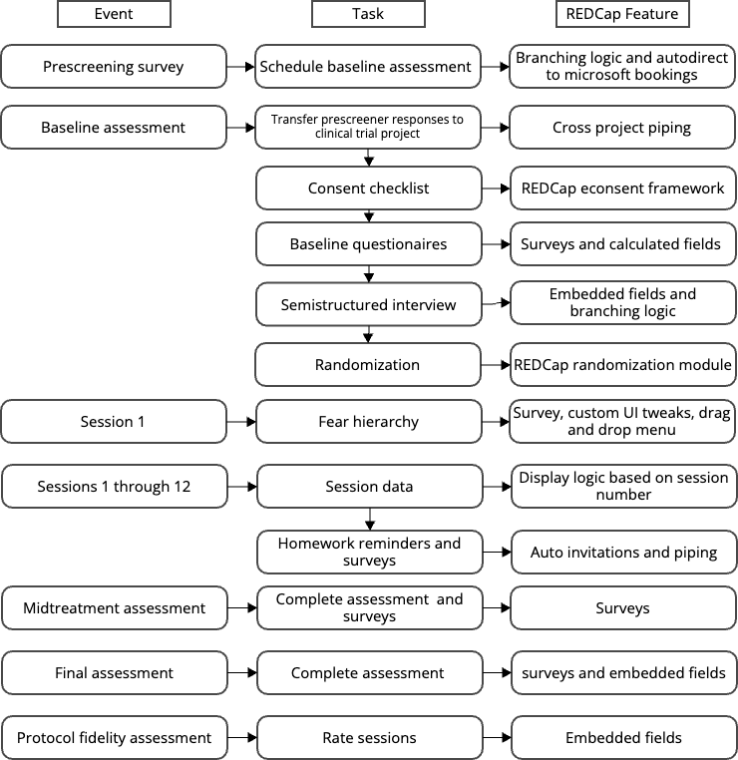
REDCap (Research Electronic Data Capture) automation and features. UI: user interface.

The second, main REDCap project will facilitate all other data collection for the feasibility trial. Participants’ online prebaseline screening questionnaire responses will be automatically transferred to the main clinical trial project using the REDCap piping function in preparation for the baseline assessment. After obtaining informed consent, supported by the REDCap e-Consent Framework feature, participants will be automatically directed to baseline questionnaire surveys, for which REDCap auto-calculates the scores. Following completion of the baseline assessment, the participant will be randomized to their respective treatment conduction using the Randomization Module. All therapy session data (ie, session number, date, time, and length), adherence checklist, clinical notes, and in-session exposure ratings will be entered into a REDCap form by the study therapist during each therapy session. Following the completion of each session, participants will receive automated survey invitations to complete homework assignments, which will be delivered on specific days during the following based on the session number, session date, and treatment condition. Weekly postsession questionnaires (ie, SIPQ and CSQ-VR) will be completed via REDCap surveys, displayed conditionally based on treatment condition (eg, the CSQ-VR will only be displayed to those in the Doxy.me VR condition). Specific REDCap automations and features used for each stage of the trial are included in Figure 2.

### Ethical Considerations

The protocol was approved by the University of South Florida institutional review board (#006215). All participant data will be stored in the University of South Florida REDCap database and only study staff members have access to the REDCap project. All data exported for analysis from REDCap will be anonymized with only participant ID numbers. Participants will be compensated US $50 for completing the baseline, midtreatment assessment, and 3-month post baseline assessment at a total of US $150. Participants will be compensated using Tango e-gift cards.

### Procedures to Ensure Participant Safety

Some questions asked during assessments and exposure exercises may cause participants distress, but this distress is expected to be similar to what participants would experience with routine care for specific fears or phobias. The study therapist will ensure that any questions causing distress are discussed with participants and provide them with techniques to reduce distress. A temporary increase in the severity of anxiety is expected at the beginning of exposure therapy, along with a gradual decline in severity over the course of treatment. If participants are distressed and unable to participate in the study following baseline assessments, or at the end of the study, participants feel that they need further treatment, the study therapist will provide a referral to therapists in the participants’ local area. Referrals will be provided verbally to participants and the study therapist will follow up in 1 week via telephone to inquire as to the state of the referral and provide further assistance as necessary. If participants endorse suicidality or homicidality at any point during the study, we will follow the standard operational procedures of the University of South Florida Department of Psychiatry and Behavioral Neurosciences requiring safety planning with the participant at risk and mandatory reporting responsibilities.

### Data Analytic Plan

We will assess the feasibility of the proposed trial methodology using the following benchmarks: (1) 30 participants will be enrolled in months 1-9 of the trial, (2) ≥70% (21/30) of participants will be retained for 3-month follow-up assessments, (3) 70% (420/600) of weekly self-report assessments will be completed, and (4) treatment fidelity we will be ≥80%. The small sample size of this feasibility trial will prevent us from making conclusions about efficacy; however, a repeated measures ANOVA will be used to conduct a preliminary assessment of between-group differences in clinical outcomes while covarying for pretreatment scores. We will also conduct a preliminary examination of associations between study targets (ie, therapeutic alliance and presence) and homework adherence. Data obtained from these analyses will inform power analyses aimed at determining sample size requirements for a fully powered efficacy trial.

## Results

**Figure 3:**
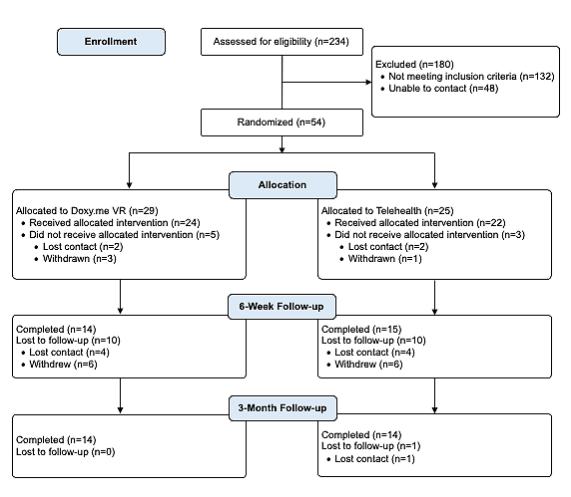
CONSORT diagram.

The first participant was enrolled on October 25, 2023, and the last therapy session and 3-month post baseline assessment were completed on July 26, 2024. In total, 54 participants were randomized. Refer to Figure 3 for the CONSORT (Consolidated Standards of Reporting Trials) diagram and Multimedia Appendix 1 for the CONSORT-EHEALTH (Consolidated Standards of Reporting Trials of Electronic and Mobile Health Applications and Online Telehealth) checklist. Data analysis for the primary and secondary aims of the clinical trial will be completed and submitted by the end of the second quarter of 2025.

## Discussion

### Overview

There will be a growing need for innovative solutions to enhance the effectiveness of remote care as TMH services continue to evolve [1,16,17]. VR has tremendous potential to increase the efficacy of evidence-based practices that were originally designed for in-person treatment but are now delivered via TMH with increased regularity (eg, exposure therapy). This feasibility trial comparing exposure therapy via Doxy.me VR versus TMH for adults with an intense fear of dogs, snakes, or spiders fills an important gap in the nascent research on telemedicine-based VR exposure therapy. Using this protocol, we will assess the feasibility of our proposed study methodology in preparation for a fully powered efficacy trial. We anticipate meeting or exceeding these feasibility criteria, which will provide valuable insights into the feasibility of conducting a large-scale efficacy trial.

### Principal Findings

As anticipated, we exceeded our recruitment goal of enrolling 30 or more participants in months 1-9 of the clinical trial by enrolling 54 participants during months 1-7. Data analysis will be completed and submitted by the second quarter of 2025 at which point we will report on the remaining feasibility results.

### Comparison With Previous Work

Our study builds upon previous research demonstrating the efficacy of VR exposure to enhance treatment delivery. While there is significant support for in-clinic VR mental health therapy, evidence for telemedicine-based VR mental health is limited. Our study addresses this important gap by evaluating the feasibility and preliminary efficacy of exposure therapy via an innovative telemedicine-based VR clinic (ie, Doxy.me VR), thereby contributing to the nascent research on the use of VR in TMH.

### Limitations

The small sample size proposed by this study limits the generalizability of our findings and prevents us from drawing conclusions about treatment efficacy. A large-scale efficacy trial will address this limitation.

### Conclusions

This feasibility randomized controlled trial comparing Doxy.me VR versus TMH represents an important step toward leveraging VR technology to enhance the delivery of evidence-based treatments via telemedicine. By evaluating the feasibility and preliminary efficacy of exposure therapy via a telemedicine-based VR clinic, we will contribute a potential solution to common barriers to remotely delivered exposure therapy.

### Future Directions

Future research will involve fully powered efficacy trials on telemedicine-based VR and the potential of this technology to improve treatment outcomes for mental health disorders.

## Data Availability

The datasets generated during and/or analyzed during this study are available from the corresponding author on reasonable request.

## Appendix

Multimedia Appendix 1 CONSORT-eHEALTH checklist (V 1.6.1).

## Abbreviations

CONSORT: Consolidated Standards of Reporting Trials
CONSORT-EHEALTH: Consolidated Standards of Reporting Trials of Electronic and Mobile Health Applications and Online Telehealth
CSQ-8: Client Satisfaction Questionnaire-8
CSQ-VR: Cybersickness Questionnaire for Virtual Reality
DART: Diagnostic Assessment Research Tool
DSM-5: Diagnostic and Statistical Manual-Fifth Edition
GAD-7: Generalized Anxiety Disorder-7
HIPAA: Health Insurance Portability and Accountability Act
PHQ-8: Patient Health Questionnaire-8
REDCap: Research Electronic Data Capture
SIPQ: Single Item Presence Questionnaire
SMSP: Severity Measure for Specific Phobias
SUS: System Usability Scale
TMH: Telemental health care
VR: virtual reality
VRET: virtual reality–based exposure therapy

## References

[1] Bunnell BE, Barrera JF, Paige SR, Turner D, Welch BM (2020). Acceptability of telemedicine features to promote its uptake in practice: a survey of community telemental health providers. Int J Environ Res Public Health.

[2] Wilczewski H, Ong T, Ivanova J, Soni H, Barrera JF, Cummins MR, Welch BM, Bunnell BE (2024). Telemedicine from home or the office: perceptions of mental health providers. Telemed J E Health.

[3] Adams SM, Rice MJ, Jones SL, Herzog E, Mackenzie LJ, Oleck LG (2018). TeleMental health: standards, reimbursement, and interstate practice [Formula: see text]. J Am Psychiatr Nurses Assoc.

[4] Flodgren G, Rachas A, Farmer AJ, Inzitari M, Shepperd S (2015). Interactive telemedicine: effects on professional practice and health care outcomes. Cochrane Database Syst Rev.

[5] Hilty DM, Ferrer DC, Parish MB, Johnston B, Callahan EJ, Yellowlees PM (2013). The effectiveness of telemental health: a 2013 review. Telemed J E Health.

[6] Yuen EK, Goetter EM, Herbert JD, Forman EM (2012). Challenges and opportunities in internet-mediated telemental health. Professional Psychology: Research and Practice.

[7] Batastini AB, Paprzycki P, Jones ACT, MacLean N (2021). Are videoconferenced mental and behavioral health services just as good as in-person? A meta-analysis of a fast-growing practice. Clin Psychol Rev.

[8] Steidtmann D, McBride S, Mishkind M (2022). Patient experiences with telemental health during the COVID-19 pandemic. J Patient Exp.

[9] Connolly SL, Miller CJ, Lindsay JA, Bauer MS (2020). A systematic review of providers' attitudes toward telemental health via videoconferencing. Clin Psychol (New York).

[10] Siegel A, Zuo Y, Moghaddamcharkari N, McIntyre RS, Rosenblat JD (2021). Barriers, benefits and interventions for improving the delivery of telemental health services during the coronavirus disease 2019 pandemic: a systematic review. Curr Opin Psychiatry.

[11] Butzner M, Cuffee Y (2021). Telehealth interventions and outcomes across rural communities in the United States: narrative review. J Med Internet Res.

[12] Baker LC, Johnson SJ, Macaulay D, Birnbaum H (2011). Integrated telehealth and care management program for medicare beneficiaries with chronic disease linked to savings. Health Aff (Millwood).

[13] Rojas SV, Gagnon M (2008). A systematic review of the key indicators for assessing telehomecare cost-effectiveness. Telemed J E Health.

[14] Wootton R, Bahaadinbeigy K, Hailey D (2011). Estimating travel reduction associated with the use of telemedicine by patients and healthcare professionals: proposal for quantitative synthesis in a systematic review. BMC Health Serv Res.

[15] Sanjula J, Katie P, Austin M, Colin M, Sarah M, Maggie J, Allison O (2023). 2023 trends shaping the health economy report. Trilliant Health.

[16] Ong T, Wilczewski H, Paige SR, Soni H, Welch BM, Bunnell BE (2021). Extended reality for enhanced telehealth during and beyond COVID-19: viewpoint. JMIR Serious Games.

[17] Bunnell BE, Kazantzis N, Paige SR, Barrera J, Thakkar RN, Turner D, Welch BM (2021). Provision of care by "Real World" telemental health providers. Front Psychol.

[18] Maples-Keller JL, Bunnell BE, Kim S, Rothbaum BO (2017). The use of virtual reality technology in the treatment of anxiety and other psychiatric disorders. Harv Rev Psychiatry.

[19] Jerdan SW, Grindle M, van Woerden HC, Kamel Boulos MN (2018). Head-mounted virtual reality and mental health: critical review of current research. JMIR Serious Games.

[20] Srivastava K, Das RC, Chaudhury S (2014). Virtual reality applications in mental health: challenges and perspectives. Ind Psychiatry J.

[21] Gagliardi M, Markowski M (2024). Active imagery rescripting in virtual reality as a promising tool to address psychological conditions. Computers in Human Behavior Reports.

[22] Bell IH, Nicholas J, Alvarez-Jimenez M, Thompson A, Valmaggia L (2020). Virtual reality as a clinical tool in mental health research and practice. Dialogues Clin Neurosci.

[23] Park MJ, Kim DJ, Lee U, Na EJ, Jeon HJ (2019). A literature overview of virtual reality (VR) in treatment of psychiatric disorders: recent advances and limitations. Front Psychiatry.

[24] Pimentel D, Foxman M, Davis DZ, Markowitz DM (2021). Virtually real, but not quite there: social and economic barriers to meeting virtual reality’s true potential for mental health. Front. Virtual Real.

[25] Goldenhersch E, Thrul J, Ungaretti J, Rosencovich N, Waitman C, Ceberio MR (2020). Virtual reality smartphone-based intervention for smoking cessation: pilot randomized controlled trial on initial clinical efficacy and adherence. J Med Internet Res.

[26] Dilgul M, Hickling LM, Antonie D, Priebe S, Bird VJ (2021). Virtual reality group therapy for the treatment of depression: a qualitative study on stakeholder perspectives. Front. Virtual Real.

[27] Tamplin J, Loveridge B, Clarke K, Li Y, J Berlowitz D (2020). Development and feasibility testing of an online virtual reality platform for delivering therapeutic group singing interventions for people living with spinal cord injury. J Telemed Telecare.

[28] Sampaio M, Haro MVN, De Sousa B, Melo WV, Hoffman HG (2021). Therapists make the switch to telepsychology to safely continue treating their patients during the COVID-19 pandemic. Virtual reality telepsychology may be next. Front Virtual Real.

[29] Pedram S, Palmisano S, Perez P, Mursic R, Farrelly M (2020). Examining the potential of virtual reality to deliver remote rehabilitation. Computers in Human Behavior.

[30] Taylor S (2003). Outcome predictors for three PTSD treatments: exposure therapy, EMDR, and relaxation training. J Cogn Psychother.

[31] Vinograd M, Craske MG (2020). Chapter 1 - history and theoretical underpinnings of exposure therapy**. Exposure Therapy for Children with Anxiety and OCD.

[32] Wells SY, Morland LA, Wilhite ER, Grubbs KM, Rauch SAM, Acierno R, McLean CP (2020). Delivering prolonged exposure therapy via videoconferencing during the COVID-19 pandemic: an overview of the research and special considerations for providers. J Trauma Stress.

[33] Schiavone E, Freeman J, O'Connor E (2021). Delivering exposure therapy via telehealth: benefits and challenges. Child Adolescent Behavior.

[34] Horigome T, Kurokawa S, Sawada K, Kudo S, Shiga K, Mimura M, Kishimoto T (2020). Virtual reality exposure therapy for social anxiety disorder: a systematic review and meta-analysis. Psychol Med.

[35] Meyerbröker K, Emmelkamp PMG (2010). Virtual reality exposure therapy in anxiety disorders: a systematic review of process-and-outcome studies. Depress Anxiety.

[36] Parsons TD, Rizzo AA (2008). Affective outcomes of virtual reality exposure therapy for anxiety and specific phobias: a meta-analysis. J Behav Ther Exp Psychiatry.

[37] Yoshinaga M, Miyazaki A, Aoki M, Ogata H, Ito Y, Hamajima T, Tokuda M, Lin L, Horigome H, Takahashi H, Nagashima M (2020). Promoting physical activity through walking to treat childhood obesity, mainly for mild to moderate obesity. Pediatr Int.

[38] Boeldt D, McMahon E, McFaul M, Greenleaf W (2019). Using virtual reality exposure therapy to enhance treatment of anxiety disorders: identifying areas of clinical adoption and potential obstacles. Front Psychiatry.

[39] Ong T, Wilczewski H, Soni H, Nisbet Q, Paige SR, Barrera JF, Welch BM, Bunnell BE (2022). The symbiosis of virtual reality exposure therapy and telemental health: a review. Front Virtual Real.

[40] Im S, Jo D, Lee SM (2024). Exploring the impact of therapeutic presence on working alliance in metaverse counseling. The Counseling Psychologist.

[41] Krogh E, Langer Á, Schmidt C (2019). Therapeutic presence: its contribution to the doctor-patient encounter. J Contin Educ Health Prof.

[42] Seuling PD, Fendel JC, Spille L, Göritz AS, Schmidt S (2024). Therapeutic alliance in videoconferencing psychotherapy compared to psychotherapy in person: a systematic review and meta-analysis. J Telemed Telecare.

[43] Buchholz JL, Abramowitz JS (2020). The therapeutic alliance in exposure therapy for anxiety-related disorders: a critical review. J Anxiety Disord.

[44] Hilty DM, Randhawa K, Maheu MM, McKean AJS, Pantera R, Mishkind MC, Rizzo AS (2020). A review of telepresence, virtual reality, and augmented reality applied to clinical care. J. technol. behav. sci.

[45] Ong T, Ivanova J, Soni H, Wilczewski H, Barrera J, Cummins M, Welch BM, Bunnell BE (2024). Therapist perspectives on telehealth-based virtual reality exposure therapy. Virtual Real.

[46] Ong T, Barrera JF, Sunkara C, Soni H, Ivanova J, Cummins MR, Schuler KR, Wilczewski H, Welch BM, Bunnell BE (2024). Mental health providers are inexperienced but interested in telehealth-based virtual reality therapy: survey study. Front Virtual Real.

[47] Harris PA, Taylor R, Minor BL, Elliott V, Fernandez M, O'Neal L, McLeod L, Delacqua G, Delacqua F, Kirby J, Duda SN, REDCap Consortium (2019). The REDCap consortium: building an international community of software platform partners. J Biomed Inform.

[48] Harris PA, Taylor R, Thielke R, Payne J, Gonzalez N, Conde JG (2009). Research electronic data capture (REDCap)--a metadata-driven methodology and workflow process for providing translational research informatics support. J Biomed Inform.

[49] Harris Paul A, Taylor Robert, Thielke Robert, Payne Jonathon, Gonzalez Nathaniel, Conde Jose G (2009). Research Electronic Data Capture (REDCap)--a metadata-driven methodology and workflow process for providing translational research informatics support. J Biomed Inform.

[50] Bouchard S, Robillard G, Larouche S, Loranger C (2012). Description of a treatment manual for in virtuo exposure with specific phobia. Virtual Reality in Psychological, Medical and Pedagogical Applications.

[51] Côté S, Bouchard S (2005). Documenting the efficacy of virtual reality exposure with psychophysiological and information processing measures. Appl Psychophysiol Biofeedback.

[52] Michaliszyn D, Marchand A, Bouchard S, Martel M, Poirier-Bisson J (2010). A randomized, controlled clinical trial of in virtuo and in vivo exposure for spider phobia. Cyberpsychol Behav Soc Netw.

[53] St-Jacques J, Bouchard S, Bélanger C (2010). Is virtual reality effective to motivate and raise interest in phobic children toward therapy? A clinical trial study of in vivo with in virtuo versus in vivo only treatment exposure. J Clin Psychiatry.

[54] Expert Clinical Trial Patient recruitment services. ClinicalConnection.

[55] ResearchMatch: The national volunteer registry. Vanderbilt University. researchmatch.

[56] Facebook.

[57] Schneider LH, Pawluk EJ, Milosevic I, Shnaider P, Rowa K, Antony MM, Musielak N, McCabe RE (2022). The diagnostic assessment research tool in action: a preliminary evaluation of a semistructured diagnostic interview for DSM-5 disorders. Psychol Assess.

[58] American Psychiatric Association (2013). Diagnostic and Statistical Manual of Mental Disorders (DSM-5 (R)).

[59] Pawluk EJ, Musielak N, Milosevic I, Rowa K, Shnaider P, Schneider LH, Antony MM, McCabe RE (2021). An evaluation of the diagnostic assessment research tool (DART) screener for DSM-5 disorders. J Psychopathol Behav Assess.

[60] Craske M, Wittchen U, Bogels S, Stein M, Andrews G, Lebeu R (2013). Severity measure for specific phobia—adult. Measurement instrument American Psychiatric Association.

[61] MacLeod S, Schneider LH, McCabe RE (2022). Investigating the psychometric properties of the severity measure for specific phobia. J Psychopathol Behav Assess.

[62] Spitzer RL, Kroenke K, Williams JBW, Löwe B (2006). A brief measure for assessing generalized anxiety disorder: the GAD-7. Arch Intern Med.

[63] Johnson SU, Ulvenes PG, Øktedalen T, Hoffart A (2019). Psychometric properties of the general anxiety disorder 7-item (GAD-7) scale in a heterogeneous psychiatric sample. Front Psychol.

[64] Kroenke K, Strine TW, Spitzer RL, Williams JBW, Berry JT, Mokdad AH (2009). The PHQ-8 as a measure of current depression in the general population. J Affect Disord.

[65] Martin A, Rief W, Klaiberg A, Braehler E (2006). Validity of the brief patient health questionnaire mood scale (PHQ-9) in the general population. Gen Hosp Psychiatry.

[66] Hatcher RL, Gillaspy JA (2006). Development and validation of a revised short version of the working alliance inventory. Psychotherapy Research.

[67] Hatcher RL, Lindqvist K, Falkenström F (2020). Psychometric evaluation of the working alliance inventory-therapist version: current and new short forms. Psychother Res.

[68] Bouchard S, Robillard G, St-Jacques J, Dumoulin S, Patry M, Renaud P (2004). Reliability and validity of a single-item measure of presence in VR.

[69] Kourtesis P, Linnell J, Amir R, Argelaguet F, MacPherson SE (2023). Cybersickness in virtual reality questionnaire (CSQ-VR): a validation and comparison against SSQ and VRSQ. Virtual Worlds.

[70] Somrak A, Pogačnik M, Guna J (2021). Suitability and comparison of questionnaires assessing virtual reality-induced symptoms and effects and user experience in virtual environments. Sensors (Basel).

[71] Larsen D L, Attkisson C C, Hargreaves W A, Nguyen T D (1979). Assessment of client/patient satisfaction: development of a general scale. Eval Program Plann.

[72] Attkisson CC, Zwick R (1982). The client satisfaction questionnaire. Psychometric properties and correlations with service utilization and psychotherapy outcome. Eval Program Plann.

[73] Peres SC, Pham T, Phillips R (2013). Validation of the system usability scale (SUS): SUS in the wild. Proc Hum Fact Ergon Soc Annu Meet.

