# Supplementary material for: Examining a Telemedicine-Based Virtual Reality Clinic in Treating Adults With Specific Phobia: Protocol for a Feasibility Randomized Controlled Efficacy Trial
Source: JMIR Res Protoc. 2025 Apr 10;14:e65770. doi: 10.2196/65770 (PMC12022526; doi:10.2196/65770)
Supplement: Multimedia Appendix 1 [file resprot_v14i1e65770_app1.pdf]

CONSORT-EHEALTH (V 1.6.1) - Submission/Publication Form

The CONSORT-EHEALTH checklist is intended for authors of randomized trials evaluating web-based and Internet-based applications/interventions, including mobile interventions, electronic games (incl multiplayer games), social media, certain telehealth applications, and other interactive and/or networked electronic applications. Some of the items (e.g. all subitems under item 5 - description of the intervention) may also be applicable for other study designs.

The goal of the CONSORT EHEALTH checklist and guideline is to be
a) a guide for reporting for authors of RCTs,
b) to form a basis for appraisal of an ehealth trial (in terms of validity)

CONSORT-EHEALTH items/subitems are MANDATORY reporting items for studies published in the Journal of Medical Internet Research and other journals / scientific societies endorsing the checklist.

Items numbered 1., 2., 3., 4a., 4b etc are original CONSORT or CONSORT-NPT (non-pharmacologic treatment) items.
Items with Roman numerals (i., ii, iii, iv etc.) are CONSORT-EHEALTH extensions/clarifications.

As the CONSORT-EHEALTH checklist is still considered in a formative stage, we would ask that you also RATE ON A SCALE OF 1-5 how important/useful you feel each item is FOR THE PURPOSE OF THE CHECKLIST and reporting guideline (optional).

Mandatory reporting items are marked with a red *.
In the textboxes, either copy & paste the relevant sections from your manuscript into this form - please include any quotes from your manuscript in QUOTATION MARKS,
or answer directly by providing additional information not in the manuscript, or elaborating on why the item was not relevant for this study.

YOUR ANSWERS WILL BE PUBLISHED AS A SUPPLEMENTARY FILE TO YOUR PUBLICATION IN JMIR AND ARE CONSIDERED PART OF YOUR PUBLICATION (IF ACCEPTED).
Please fill in these questions diligently. Information will not be copyedited, so please use proper spelling and grammar, use correct capitalization, and avoid abbreviations.

DO NOT FORGET TO SAVE AS PDF _AND_ CLICK THE SUBMIT BUTTON SO YOUR ANSWERS ARE IN OUR DATABASE !!!

Citation Suggestion (if you append the pdf as Appendix we suggest to cite this paper in the caption):
Eysenbach G, CONSORT-EHEALTH Group

CONSORT-EHEALTH: Improving and Standardizing Evaluation Reports of Web-based and Mobile Health Interventions
J Med Internet Res 2011;13(4):e126
URL: http://www.jmir.org/2011/4/e126/
doi: 10.2196/jmir.1923
PMID: 22209829

kaitlynritaschuler@gmail.com Switch account

Not shared

Draft not saved

* Indicates required question

Your name *

First Last

Kaitlyn Schuler

Primary Affiliation (short), City, Country *

University of Toronto, Toronto, Canada

University of North Carolina at Wilmington, U.S

Your e-mail address *

abc@gmail.com

schulerka@uncw.edu

Title of your manuscript *

Provide the (draft) title of your manuscript.

Examining the Efficacy of a Telemedicine-Based Virtual Reality Clinic in Treating Adults with Specific Phobia: Protocol for A Feasibility Randomized Controlled Efficacy Trial

Name of your App/Software/Intervention *

If there is a short and a long/alternate name, write the short name first and add the long name in brackets.

Doxy.me VR

Evaluated Version (if any)

e.g. "V1", "Release 2017-03-01", "Version 2.0.27913"

Only one version of the Doxy.me VR clinic exist

Language(s) *

What language is the intervention/app in? If multiple languages are available, separate by comma (e.g. "English, French")

English

URL of your Intervention Website or App

e.g. a direct link to the mobile app on app in appstore (itunes, Google Play), or URL of the website. If the intervention is a DVD or hardware, you can also link to an Amazon page.

https://www.meta.com/experiences/doxyme-vr/6920571221312916/?srsltid=AfmBOor0Clcv

URL of an image/screenshot (optional)

https://www.meta.com/experiences/doxyme-vr/6920571221312916/?srsltid=AfmBOor0Clcv

Accessibility *

Can an enduser access the intervention presently?

- (•) access is free and open
- ( ) access only for special usergroups, not open
- ( ) access is open to everyone, but requires payment/subscription/in-app purchases
- ( ) app/intervention no longer accessible
- ( ) Other:

Primary Medical Indication/Disease/Condition *

e.g. "Stress", "Diabetes", or define the target group in brackets after the condition, e.g. "Autism (Parents of children with)", "Alzheimers (Informal Caregivers of)"

Animal Phobia

Primary Outcomes measured in trial *

comma-separated list of primary outcomes reported in the trial

specific phobia, anxiety, and depression sympt

Secondary/other outcomes

Are there any other outcomes the intervention is expected to affect?

therapeutic alliance and presence

Recommended "Dose" *

What do the instructions for users say on how often the app should be used?

- (x) Approximately Daily
- ( ) Approximately Weekly
- ( ) Approximately Monthly
- ( ) Approximately Yearly
- ( ) "as needed"
- ( ) Other:

Approx. Percentage of Users (starters) still using the app as recommended after 3 months *

- (x) unknown / not evaluated
- ( ) 0-10%
- ( ) 11-20%
- ( ) 21-30%
- ( ) 31-40%
- ( ) 41-50%
- ( ) 51-60%
- ( ) 61-70%
- ( ) 71%-80%
- ( ) 81-90%
- ( ) 91-100%
- ( ) Other:

Overall, was the app/intervention effective? *

- ○ yes: all primary outcomes were significantly better in intervention group vs control
- ○ partly: SOME primary outcomes were significantly better in intervention group vs control
- ○ no statistically significant difference between control and intervention
- ○ potentially harmful: control was significantly better than intervention in one or more outcomes
- ◉ inconclusive: more research is needed
- ○ Other:

Article Preparation Status/Stage *

At which stage in your article preparation are you currently (at the time you fill in this form)

- ○ not submitted yet - in early draft status
- ○ not submitted yet - in late draft status, just before submission
- ○ submitted to a journal but not reviewed yet
- ○ submitted to a journal and after receiving initial reviewer comments
- ○ submitted to a journal and accepted, but not published yet
- ○ published
- ◉ Other: Accepted, completing minor revisions

Journal *

If you already know where you will submit this paper (or if it is already submitted), please provide the journal name (if it is not JMIR, provide the journal name under "other")

- ○ not submitted yet / unclear where I will submit this
- ○ Journal of Medical Internet Research (JMIR)
- ○ JMIR mHealth and UHealth
- ○ JMIR Serious Games
- ○ JMIR Mental Health
- ○ JMIR Public Health
- ○ JMIR Formative Research
- ◉ Other JMIR sister journal
- ○ Other:

Is this a full powered effectiveness trial or a pilot/feasibility trial? *

- ◉ Pilot/feasibility
- ○ Fully powered

Manuscript tracking number *

If this is a JMIR submission, please provide the manuscript tracking number under "other" (The ms tracking number can be found in the submission acknowledgement email, or when you login as author in JMIR. If the paper is already published in JMIR, then the ms tracking number is the four-digit number at the end of the DOI, to be found at the bottom of each published article in JMIR)

- ○ no ms number (yet) / not (yet) submitted to / published in JMIR
- ◉ Other: 65770

TITLE AND ABSTRACT

1a) TITLE: Identification as a randomized trial in the title

1a) Does your paper address CONSORT item 1a? *

I.e does the title contain the phrase "Randomized Controlled Trial"? (if not, explain the reason under "other")

- (●) yes
- ( ) Other:

1a-i) Identify the mode of delivery in the title

Identify the mode of delivery. Preferably use “web-based” and/or “mobile” and/or “electronic game” in the title. Avoid ambiguous terms like “online”, “virtual”, “interactive”. Use “Internet-based” only if Intervention includes non-web-based Internet components (e.g. email), use “computer-based” or “electronic” only if offline products are used. Use “virtual” only in the context of “virtual reality” (3-D worlds). Use “online” only in the context of “online support groups”. Complement or substitute product names with broader terms for the class of products (such as “mobile” or “smart phone” instead of “iphone”), especially if the application runs on different platforms.

	1	2	3	4	5	
subitem not at all important	○	○	○	○	●	essential

Clear selection

Does your paper address subitem 1a-i? *

Copy and paste relevant sections from manuscript title (include quotes in quotation marks "like this" to indicate direct quotes from your manuscript), or elaborate on this item by providing additional information not in the ms, or briefly explain why the item is not applicable/relevant for your study

Examining the Efficacy of a Telemedicine-Based Virtual Reality Clinic in Treating Adults with Specific Phobia: Protocol for A Feasibility Randomized Controlled Efficacy Trial

1a-ii) Non-web-based components or important co-interventions in title

Mention non-web-based components or important co-interventions in title, if any (e.g., “with telephone support”).

	1	2	3	4	5	
subitem not at all important	○	○	○	○	◉	essential

Clear selection

Does your paper address subitem 1a-ii?

Copy and paste relevant sections from manuscript title (include quotes in quotation marks "like this" to indicate direct quotes from your manuscript), or elaborate on this item by providing additional information not in the ms, or briefly explain why the item is not applicable/relevant for your study

"Examining the Efficacy of a Telemedicine-Based Virtual Reality Clinic in Treating Adults with Specific Phobia: Protocol for A Feasibility Randomized Controlled Efficacy Trial " The entire clinical trial was conducted over tele-health.

1a-iii) Primary condition or target group in the title

Mention primary condition or target group in the title, if any (e.g., “for children with Type I Diabetes”) Example: A Web-based and Mobile Intervention with Telephone Support for Children with Type I Diabetes: Randomized Controlled Trial

	1	2	3	4	5	
subitem not at all important	○	○	○	○	◉	essential

Clear selection

Does your paper address subitem 1a-iii? *

Copy and paste relevant sections from manuscript title (include quotes in quotation marks "like this" to indicate direct quotes from your manuscript), or elaborate on this item by providing additional information not in the ms, or briefly explain why the item is not applicable/relevant for your study

Examining the Efficacy of a Telemedicine-Based Virtual Reality Clinic in Treating Adults with Specific Phobia: Protocol for A Feasibility Randomized Controlled Efficacy Trial

1b) ABSTRACT: Structured summary of trial design, methods, results, and conclusions

NPT extension: Description of experimental treatment, comparator, care providers, centers, and blinding status.

1b-i) Key features/functionalities/components of the intervention and comparator in the METHODS section of the ABSTRACT

Mention key features/functionalities/components of the intervention and comparator in the abstract. If possible, also mention theories and principles used for designing the site. Keep in mind the needs of systematic reviewers and indexers by including important synonyms. (Note: Only report in the abstract what the main paper is reporting. If this information is missing from the main body of text, consider adding it)

	1	2	3	4	5	
subitem not at all important	○	○	◉	○	○	essential

Clear selection

Does your paper address subitem 1b-i? *

Copy and paste relevant sections from the manuscript abstract (include quotes in quotation marks "like this" to indicate direct quotes from your manuscript), or elaborate on this item by providing additional information not in the ms, or briefly explain why the item is not applicable/relevant for your study

"This feasibility randomized controlled efficacy trial aims to fill both of these gaps by piloting a novel telemedicine-based VR application (Doxy.me VR) equipped with animal phobia exposure stimuli."

1b-ii) Level of human involvement in the METHODS section of the ABSTRACT

Clarify the level of human involvement in the abstract, e.g., use phrases like “fully automated” vs. “therapist/nurse/care provider/physician-assisted” (mention number and expertise of providers involved, if any). (Note: Only report in the abstract what the main paper is reporting. If this information is missing from the main body of text, consider adding it)

	1	2	3	4	5	
subitem not at all important	○	○	○	○	◉	essential

Clear selection

Does your paper address subitem 1b-ii?

Copy and paste relevant sections from the manuscript abstract (include quotes in quotation marks "like this" to indicate direct quotes from your manuscript), or elaborate on this item by providing additional information not in the ms, or briefly explain why the item is not applicable/relevant for your study

All procedures will be partially automated using REDCap forms and surveys, but the baseline assessment of phobia symptoms will be administered by the study therapist.

1b-iii) Open vs. closed, web-based (self-assessment) vs. face-to-face assessments in the METHODS section of the ABSTRACT

Mention how participants were recruited (online vs. offline), e.g., from an open access website or from a clinic or a closed online user group (closed usergroup trial), and clarify if this was a purely web-based trial, or there were face-to-face components (as part of the intervention or for assessment). Clearly say if outcomes were self-assessed through questionnaires (as common in web-based trials). Note: In traditional offline trials, an open trial (open-label trial) is a type of clinical trial in which both the researchers and participants know which treatment is being administered. To avoid confusion, use “blinded” or “unblinded” to indicated the level of blinding instead of “open”, as “open” in web-based trials usually refers to “open access” (i.e. participants can self-enrol). (Note: Only report in the abstract what the main paper is reporting. If this information is missing from the main body of text, consider adding it)

	1	2	3	4	5	
subitem not at all important	○	○	○	○	●	essential

Clear selection

Does your paper address subitem 1b-iii?

Copy and paste relevant sections from the manuscript abstract (include quotes in quotation marks "like this" to indicate direct quotes from your manuscript), or elaborate on this item by providing additional information not in the ms, or briefly explain why the item is not applicable/relevant for your study

The abstract explicitly states that all procedures are complete over tele-health.

1b-iv) RESULTS section in abstract must contain use data

Report number of participants enrolled/assessed in each group, the use/uptake of the intervention (e.g., attrition/adherence metrics, use over time, number of logins etc.), in addition to primary/secondary outcomes. (Note: Only report in the abstract what the main paper is reporting. If this information is missing from the main body of text, consider adding it)

	1	2	3	4	5	
subitem not at all important	○	○	○	○	◉	essential

Clear selection

Does your paper address subitem 1b-iv?

Copy and paste relevant sections from the manuscript abstract (include quotes in quotation marks "like this" to indicate direct quotes from your manuscript), or elaborate on this item by providing additional information not in the ms, or briefly explain why the item is not applicable/relevant for your study

"In total, 54 participants were enrolled, randomized, and treated between October 25th 2023 and July 26th 2024 (Doxy.me VR, n = 28, TMH, n= 26). Data analysis for the primary and secondary aims of the clinical trial will be completed and submitted by the end of the second quarter of 2025. " The remaining metrics will be reported after data analysis is completed.

1b-v) CONCLUSIONS/DISCUSSION in abstract for negative trials

Conclusions/Discussions in abstract for negative trials: Discuss the primary outcome - if the trial is negative (primary outcome not changed), and the intervention was not used, discuss whether negative results are attributable to lack of uptake and discuss reasons. (Note: Only report in the abstract what the main paper is reporting. If this information is missing from the main body of text, consider adding it)

	1	2	3	4	5	
subitem not at all important	◉	○	○	○	○	essential

Clear selection

Does your paper address subitem 1b-v?

Copy and paste relevant sections from the manuscript abstract (include quotes in quotation marks "like this" to indicate direct quotes from your manuscript), or elaborate on this item by providing additional information not in the ms, or briefly explain why the item is not applicable/relevant for your study

This item is not applicable because this is a protocol paper

INTRODUCTION

2a) In INTRODUCTION: Scientific background and explanation of rationale

2a-i) Problem and the type of system/solution

Describe the problem and the type of system/solution that is object of the study: intended as stand-alone intervention vs. incorporated in broader health care program? Intended for a particular patient population? Goals of the intervention, e.g., being more cost-effective to other interventions, replace or complement other solutions? (Note: Details about the intervention are provided in “Methods” under 5)

	1	2	3	4	5	
subitem not at all important	○	○	○	○	◉	essential

Clear selection

Does your paper address subitem 2a-i? *

Copy and paste relevant sections from the manuscript (include quotes in quotation marks "like this" to indicate direct quotes from your manuscript), or elaborate on this item by providing additional information not in the ms, or briefly explain why the item is not applicable/relevant for your study

"Telemental health care (TMH) has revolutionized mental health services by providing accessible, personalized treatment from the comfort and privacy of home. [1,2] TMH is equally to more effective than in-person care, with patients generally reporting higher satisfaction and reduced costs.[3–11]Further, TMH alleviates socio-cultural (e.g., stigma) and geographic (e.g., transportation) barriers to mental healthcare. [12–14] TMH increased from 41.8% to 62.8% of all tele-health services during 2022, surpassing all other forms of virtual clinical care. [15] This trend suggests that clients view TMH as an appropriate substitute for in-person care. As TMH services continue to evolve, there will be a growing need for innovative solutions to enhance the effectiveness of remote care. [1,16,17]"

2a-ii) Scientific background, rationale: What is known about the (type of) system

Scientific background, rationale: What is known about the (type of) system that is the object of the study (be sure to discuss the use of similar systems for other conditions/diagnoses, if appropiate), motivation for the study, i.e. what are the reasons for and what is the context for this specific study, from which stakeholder viewpoint is the study performed, potential impact of findings [2]. Briefly justify the choice of the comparator.

	1	2	3	4	5	
subitem not at all important	○	○	○	○	◉	essential

Clear selection

Does your paper address subitem 2a-ii? *

Copy and paste relevant sections from the manuscript (include quotes in quotation marks "like this" to indicate direct quotes from your manuscript), or elaborate on this item by providing additional information not in the ms, or briefly explain why the item is not applicable/relevant for your study

"Telemental health care (TMH) has revolutionized mental health services by providing accessible, personalized treatment from the comfort and privacy of home. [1,2] TMH is equally to more effective than in-person care, with patients generally reporting higher satisfaction and reduced costs.[3–11]Further, TMH alleviates socio-cultural (e.g., stigma) and geographic (e.g., transportation) barriers to mental healthcare. [12–14] TMH increased from 41.8% to 62.8% of all tele-health services during 2022, surpassing all other forms of virtual clinical care. [15] This trend suggests that clients view TMH as an appropriate substitute for in-person care. As TMH services continue to evolve, there will be a growing need for innovative solutions to enhance the effectiveness of remote care. [1,16,17]"

2b) In INTRODUCTION: Specific objectives or hypotheses

Does your paper address CONSORT subitem 2b? *

Copy and paste relevant sections from the manuscript (include quotes in quotation marks "like this" to indicate direct quotes from your manuscript), or elaborate on this item by providing additional information not in the ms, or briefly explain why the item is not applicable/relevant for your study

"We present the protocol for a feasibility randomized controlled efficacy trial comparing exposure therapy delivered via Doxy.me VR versus standard TMH to adults with an intense fear of dogs, snakes, and/or spiders. The primary aim of this study is to assess the feasibility of and refine our study methodology in preparation for a large, fully-powered randomized controlled efficacy trial. The secondary aim is to conduct a preliminary examination of clinical outcomes, including between-group differences in specific phobia symptoms, therapeutic alliance, and presence. "

METHODS

3a) Description of trial design (such as parallel, factorial) including allocation ratio

Does your paper address CONSORT subitem 3a? *

Copy and paste relevant sections from the manuscript (include quotes in quotation marks "like this" to indicate direct quotes from your manuscript), or elaborate on this item by providing additional information not in the ms, or briefly explain why the item is not applicable/relevant for your study

This study will use a feasibility randomized controlled efficacy trial design. This single-site trial will enroll a maximum of 60 adults with self-reported fear of dogs, snakes, and/or spiders with the goal of completing treatment with 30 adults. Participants will be randomly assigned using the REDCap[47,48] Randomization Module on a 1:1 allocation ratio to receive 12 weekly sessions of exposure therapy over the course of three months delivered via standard TMH (n=15) vs. Doxy.me VR (n=15). Participants randomized to the Doxy.me group will be provided with Meta Quest 2 VR headsets preloaded with the Doxy.me VR application, to be returned to the study team following completion of the study. All therapy sessions will be conducted via remote videoconferencing, with the Doxy.me VR group transitioning to the Doxy.me VR clinic for the exposure exercise portion of the session, and the standard TMH group using multimedia (i.e., photos, videos) shared by the study therapist during video calls.

3b) Important changes to methods after trial commencement (such as eligibility criteria), with reasons

Does your paper address CONSORT subitem 3b? *

Copy and paste relevant sections from the manuscript (include quotes in quotation marks "like this" to indicate direct quotes from your manuscript), or elaborate on this item by providing additional information not in the ms, or briefly explain why the item is not applicable/relevant for your study

There were no important changes to the methods after the trial began.

3b-i) Bug fixes, Downtimes, Content Changes

Bug fixes, Downtimes, Content Changes: ehealth systems are often dynamic systems. A description of changes to methods therefore also includes important changes made on the intervention or comparator during the trial (e.g., major bug fixes or changes in the functionality or content) (5-iii) and other “unexpected events” that may have influenced study design such as staff changes, system failures/downtimes, etc. [2].

	1	2	3	4	5	
subitem not at all important	(selected)					essential

Clear selection

Does your paper address subitem 3b-i?

Copy and paste relevant sections from the manuscript (include quotes in quotation marks "like this" to indicate direct quotes from your manuscript), or elaborate on this item by providing additional information not in the ms, or briefly explain why the item is not applicable/relevant for your study

There were no substantial changes made to the VR clinic throughout the clinical trial.

4a) Eligibility criteria for participants

Does your paper address CONSORT subitem 4a? *

Copy and paste relevant sections from the manuscript (include quotes in quotation marks "like this" to indicate direct quotes from your manuscript), or elaborate on this item by providing additional information not in the ms, or briefly explain why the item is not applicable/relevant for your study

Eligible participants will: (1) be ≥ 18 years old; (2) have a self-reported fear of dogs, snakes, and/or spiders; (3) have Subthreshold or Present Specific Phobia symptoms as determined by the study therapist via administration of the Diagnostic Assessment Research Tool (DART) Specific Phobia Module[53]; (4) have access to the internet and a computer or smartphone with video conferencing capabilities; and (4) plan to reside in the state of Florida for the duration of the study.

An individual who endorses any of the following criteria will be excluded from participation in the study: (1) participation in ongoing mental health therapy from a non-study therapist; (2) changes to psychotropic medication use within six weeks preceding enrollment in the trial; (3) active suicidal and/or homicidal intent or plan as determined by the study therapist via the DART Risk Assessment Module[53]; (4) active auditory, visual, and/or tactile hallucinations via the DART Psychosis Module screening question; or (5) a diagnosis of photosensitive epilepsy by a medical doctor or a history of experiencing seizures caused by photosensitivity.

4a-i) Computer / Internet literacy

Computer / Internet literacy is often an implicit “de facto” eligibility criterion - this should be explicitly clarified.

	1	2	3	4	5	
subitem not at all important	○	○	○	○	◉	essential

Clear selection

Does your paper address subitem 4a-i?

Copy and paste relevant sections from the manuscript (include quotes in quotation marks "like this" to indicate direct quotes from your manuscript), or elaborate on this item by providing additional information not in the ms, or briefly explain why the item is not applicable/relevant for your study

Having access to the internet and a computer or smartphone with video conferencing capabilities was a criterion. We included no other measure of computer/internet literacy.

4a-ii) Open vs. closed, web-based vs. face-to-face assessments:

Open vs. closed, web-based vs. face-to-face assessments: Mention how participants were recruited (online vs. offline), e.g., from an open access website or from a clinic, and clarify if this was a purely web-based trial, or there were face-to-face components (as part of the intervention or for assessment), i.e., to what degree got the study team to know the participant. In online-only trials, clarify if participants were quasi-anonymous and whether having multiple identities was possible or whether technical or logistical measures (e.g., cookies, email confirmation, phone calls) were used to detect/prevent these.

	1	2	3	4	5	
subitem not at all important	○	○	○	○	◉	essential

Clear selection

Does your paper address subitem 4a-ii? *

Copy and paste relevant sections from the manuscript (include quotes in quotation marks "like this" to indicate direct quotes from your manuscript), or elaborate on this item by providing additional information not in the ms, or briefly explain why the item is not applicable/relevant for your study

Potential participants will be recruited through Clinical Connection, Research Match, Facebook advertisements, and flyers distributed on the University of South Florida campus and off campus community centers. Potential participants will be provided with a URL or QR code directing them to an Online Pre-Baseline Screening Questionnaire administered via REDCap. Those meeting preliminary eligibility criteria will then schedule an initial consent and baseline assessment visit via Microsoft Bookings with text/email reminders 24 hours and one hour prior to the scheduled visit time.

4a-iii) Information giving during recruitment

Information given during recruitment. Specify how participants were briefed for recruitment and in the informed consent procedures (e.g., publish the informed consent documentation as appendix, see also item X26), as this information may have an effect on user self-selection, user expectation and may also bias results.

	1	2	3	4	5	
subitem not at all important	○	○	○	○	◉	essential

Clear selection

Does your paper address subitem 4a-iii?

Copy and paste relevant sections from the manuscript (include quotes in quotation marks "like this" to indicate direct quotes from your manuscript), or elaborate on this item by providing additional information not in the ms, or briefly explain why the item is not applicable/relevant for your study

"The consent and baseline assessment visit will be conducted via video-conferencing platform and will not be audio or video recorded. During this visit, study staff will (1) confirm the potential participant's responses on the Online Pre-Screening Questionnaire; (2) provide detailed information about the study and obtain informed consent; (3) assist participants in completing baseline questionnaires via REDCap survey; (4) administer the Specific Phobia and Risk Assessment modules of the Diagnostic Assessment Research Tool (DART); and (5) make a final determination on the participant's eligibility. Eligible participants will then be randomized using the REDCap Randomization Module and scheduled for their first treatment visit. "

4b) Settings and locations where the data were collected

Does your paper address CONSORT subitem 4b? *

Copy and paste relevant sections from the manuscript (include quotes in quotation marks "like this" to indicate direct quotes from your manuscript), or elaborate on this item by providing additional information not in the ms, or briefly explain why the item is not applicable/relevant for your study

We specify the university at which the study was conducted and that study sessions and assessments were done over TMH

4b-i) Report if outcomes were (self-)assessed through online questionnaires

Clearly report if outcomes were (self-)assessed through online questionnaires (as common in web-based trials) or otherwise.

	1	2	3	4	5	
subitem not at all important	○	○	○	○	◉	essential

Clear selection

Does your paper address subitem 4b-i? *

Copy and paste relevant sections from the manuscript (include quotes in quotation marks "like this" to indicate direct quotes from your manuscript), or elaborate on this item by providing additional information not in the ms, or briefly explain why the item is not applicable/relevant for your study

"All assessments were chosen considering several factors including: (1) sound psychometric properties, (2) ease of administration, (3) past use in similar clinical trials, and (4) brevity. All assessments will be facilitated remotely by the study therapist and will occur at baseline, each session, mid-treatment (i.e., after completion of six therapy sessions, up to six-weeks post-baseline), and 12-weeks post-baseline. Self-report questionnaires will be completed by patients via REDCap survey with the study therapist present to answer any questions. Structured diagnostic interviews will be administered by study therapists and recorded in REDCap. "

4b-ii) Report how institutional affiliations are displayed

Report how institutional affiliations are displayed to potential participants [on ehealth media], as affiliations with prestigious hospitals or universities may affect volunteer rates, use, and reactions with regards to an intervention.(Not a required item – describe only if this may bias results)

	1	2	3	4	5	
subitem not at all important	○	○	◉	○	○	essential

Clear selection

Does your paper address subitem 4b-ii?

Copy and paste relevant sections from the manuscript (include quotes in quotation marks "like this" to indicate direct quotes from your manuscript), or elaborate on this item by providing additional information not in the ms, or briefly explain why the item is not applicable/relevant for your study

Participants were informed during the informed consent that the study team was at USF.

5) The interventions for each group with sufficient details to allow replication, including how and when they were actually administered

5-i) Mention names, credential, affiliations of the developers, sponsors, and owners

Mention names, credential, affiliations of the developers, sponsors, and owners [6] (if authors/evaluators are owners or developer of the software, this needs to be declared in a “Conflict of interest” section or mentioned elsewhere in the manuscript).

	1	2	3	4	5	
subitem not at all important	○	○	○	○	◉	essential

Clear selection

Does your paper address subitem 5-i?

Copy and paste relevant sections from the manuscript (include quotes in quotation marks "like this" to indicate direct quotes from your manuscript), or elaborate on this item by providing additional information not in the ms, or briefly explain why the item is not applicable/relevant for your study

"Drs. Bunnell, Ong, and Welch are employees of Doxy.me Inc., a commercial telemedicine company. The authors declare no other conflicts of interest. "

5-ii) Describe the history/development process

Describe the history/development process of the application and previous formative evaluations (e.g., focus groups, usability testing), as these will have an impact on adoption/use rates and help with interpreting results.

	1	2	3	4	5	
subitem not at all important	○	○	○	○	◉	essential

Clear selection

Does your paper address subitem 5-ii?

Copy and paste relevant sections from the manuscript (include quotes in quotation marks "like this" to indicate direct quotes from your manuscript), or elaborate on this item by providing additional information not in the ms, or briefly explain why the item is not applicable/relevant for your study

"To explore the potential of telemedicine-based VR therapy, we conducted in-depth interviews with 18 practicing TMH therapists who had delivered exposure therapy via conventional telemedicine.[45] Therapists wanted interactive VR features to build rapport with their patients and the ability to customize VR components for individual patient needs. Therapists needed clinical evidence supporting tele-VR, low costs to adopt VR into their TMH practice, and information on possible side effects for patients. We then surveyed 176 practicing TMH therapists about their perspectives on telemedicine-based VR. We asked therapists to rate the relative importance of tele-VR simulations, features, and implementation factors. Therapists strongly favored tele-VR simulations related to social anxiety, flying, driving, and animals. Therapists rated between-session VR exercises and immersive cooperative activities as some of their most valued tele-VR features. Therapists' highest rated factors for implementing telemedicine-based VR was HIPAA compliance, followed by affordability, insurance coverage, and accessibility. [46] "

5-iii) Revisions and updating

Revisions and updating. Clearly mention the date and/or version number of the application/intervention (and comparator, if applicable) evaluated, or describe whether the intervention underwent major changes during the evaluation process, or whether the development and/or content was "frozen" during the trial. Describe dynamic components such as news feeds or changing content which may have an impact on the replicability of the intervention (for unexpected events see item 3b).

	1	2	3	4	5	
subitem not at all important	◉	○	○	○	○	essential

Clear selection

Does your paper address subitem 5-iii?

Copy and paste relevant sections from the manuscript (include quotes in quotation marks "like this" to indicate direct quotes from your manuscript), or elaborate on this item by providing additional information not in the ms, or briefly explain why the item is not applicable/relevant for your study

There were no major changes to the VR clinic throughout the clinical trial and there remains only one version of the clinic.

5-iv) Quality assurance methods

Provide information on quality assurance methods to ensure accuracy and quality of information provided [1], if applicable.

	1	2	3	4	5	
subitem not at all important	(●)	( )	( )	( )	( )	essential

Clear selection

Does your paper address subitem 5-iv?

Copy and paste relevant sections from the manuscript (include quotes in quotation marks "like this" to indicate direct quotes from your manuscript), or elaborate on this item by providing additional information not in the ms, or briefly explain why the item is not applicable/relevant for your study

The study therapist review all self-report measures with the participants before enrollment to ensure accuracy of the data. There was no missing data as all responses were required on the REDCap forms.

5-v) Ensure replicability by publishing the source code, and/or providing screenshots/screen-capture video, and/or providing flowcharts of the algorithms used

Ensure replicability by publishing the source code, and/or providing screenshots/screen-capture video, and/or providing flowcharts of the algorithms used. Replicability (i.e., other researchers should in principle be able to replicate the study) is a hallmark of scientific reporting.

	1	2	3	4	5	
subitem not at all important	○	○	○	○	◉	essential

Clear selection

Does your paper address subitem 5-v?

Copy and paste relevant sections from the manuscript (include quotes in quotation marks "like this" to indicate direct quotes from your manuscript), or elaborate on this item by providing additional information not in the ms, or briefly explain why the item is not applicable/relevant for your study

"The data sets generated during and/or analyzed during this study are available from the corresponding author on reasonable request." Code and prototypes for the VR clinic will not be made publicly available as they belong to Doxy.me; however, we are including screenshots of the clinic in the manuscript.

5-vi) Digital preservation

Digital preservation: Provide the URL of the application, but as the intervention is likely to change or disappear over the course of the years; also make sure the intervention is archived (Internet Archive, webcitation.org, and/or publishing the source code or screenshots/videos alongside the article). As pages behind login screens cannot be archived, consider creating demo pages which are accessible without login.

	1	2	3	4	5	
subitem not at all important	○	○	○	○	◉	essential

Clear selection

Does your paper address subitem 5-vi?

Copy and paste relevant sections from the manuscript (include quotes in quotation marks "like this" to indicate direct quotes from your manuscript), or elaborate on this item by providing additional information not in the ms, or briefly explain why the item is not applicable/relevant for your study

https://www.meta.com/experiences/doxyme-vr/6920571221312916/?srsltid=AfmBOor0ClcwqeG25RhA_yxaGecrP-bY8KZuKU4BAn_uUj4HIPtGKaaa

5-vii) Access

Access: Describe how participants accessed the application, in what setting/context, if they had to pay (or were paid) or not, whether they had to be a member of specific group. If known, describe how participants obtained "access to the platform and Internet" [1]. To ensure access for editors/reviewers/readers, consider to provide a "backdoor" login account or demo mode for reviewers/readers to explore the application (also important for archiving purposes, see vi).

	1	2	3	4	5	
subitem not at all important	○	○	○	○	◉	essential

Clear selection

Does your paper address subitem 5-vii? *

Copy and paste relevant sections from the manuscript (include quotes in quotation marks "like this" to indicate direct quotes from your manuscript), or elaborate on this item by providing additional information not in the ms, or briefly explain why the item is not applicable/relevant for your study

Participants were mailed headsets with the clinic pre-downloaded. "To join the VR session, the therapist provides their unique, persistent 4-digit room code to their client, who enters the code to check in." and "Participants randomized to Doxy.me VR received a Meta Quest 2 headset via mail with the VR clinic already downloaded."

5-viii) Mode of delivery, features/functionalities/components of the intervention and comparator, and the theoretical framework

Describe mode of delivery, features/functionalities/components of the intervention and comparator, and the theoretical framework [6] used to design them (instructional strategy [1], behaviour change techniques, persuasive features, etc., see e.g., [7, 8] for terminology). This includes an in-depth description of the content (including where it is coming from and who developed it) [1]," whether [and how] it is tailored to individual circumstances and allows users to track their progress and receive feedback" [6]. This also includes a description of communication delivery channels and – if computer-mediated communication is a component – whether communication was synchronous or asynchronous [6]. It also includes information on presentation strategies [1], including page design principles, average amount of text on pages, presence of hyperlinks to other resources, etc. [1].

	1	2	3	4	5	
subitem not at all important	○	○	○	○	◉	essential

Clear selection

Does your paper address subitem 5-viii? *

Copy and paste relevant sections from the manuscript (include quotes in quotation marks "like this" to indicate direct quotes from your manuscript), or elaborate on this item by providing additional information not in the ms, or briefly explain why the item is not applicable/relevant for your study

This is a description of the VR clinic itself: "Doxy.me VR is designed for a therapist to meet with a client remotely in a private, comfortable VR clinic that looks and feels like a therapist's office (see Figure 1 for screenshots of the clinic). To join the VR session, the therapist provides their unique, persistent 4-digit room code to their client, who enters the code to check in. Once the therapist admits the client, they can interact by speaking and gesturing with each other in immersive VR. Therapists can use a menu to spawn animals such as dogs, snakes, and spiders for use in treating specific phobias. Multiple exemplars are available for each type of animal in varying sizes. For example, there are small dogs (e.g., Chihuahua, Corgi, Jack Russell Terrier), medium dogs (e.g., Golden Retriever, Shiba Inu, Pit Bull), and large dogs (e.g., German Shepherd, Doberman, Husky). Each animal is animated with four behavior states: idle (i.e., no movement), calm (i.e., small, slow movements in a relaxed posture), active (i.e., fast, frequent movements in a playful posture), and aggressive (i.e., fast, attacking movements in a defensive posture). Each behavior state also includes corresponding audio such as a calm dog breathing lightly, an active dog panting and barking playfully, and an aggressive dog snarling and barking loudly. Therapists can select, rotate, and move these animals around the room before making them visible to clients. Once therapists make the animal visible, they can continue to move and rotate the animal or remove the animal entirely. Clients cannot directly manipulate objects; however, clients gain access to all the control features while engaging with Doxy.me VR's homework mode, which is used for between-session practice. "

5-ix) Describe use parameters

Describe use parameters (e.g., intended "doses" and optimal timing for use). Clarify what instructions or recommendations were given to the user, e.g., regarding timing, frequency, heaviness of use, if any, or was the intervention used ad libitum.

	1	2	3	4	5	
subitem not at all important	○	○	○	○	◉	essential

Clear selection

Does your paper address subitem 5-ix?

Copy and paste relevant sections from the manuscript (include quotes in quotation marks "like this" to indicate direct quotes from your manuscript), or elaborate on this item by providing additional information not in the ms, or briefly explain why the item is not applicable/relevant for your study

The present study will follow the treatment protocol developed specifically for VRET by Bouchard et al. [49–52] Following the baseline assessment session, treatment consists of 12, ≤ 60-minute therapy sessions, including one psychoeducation and treatment planning session (session 1), 10 exposure sessions (sessions 2-11), and one relapse prevention session (session 12). During the first therapy session, patients learn about the principles of Cognitive Behavioral Therapy (CBT), the etiology of anxiety and specific phobias, and procedures for exposure. They then complete a fear hierarchy (i.e., a list of situations ranked from least to most anxiety provoking) with therapist guidance. Fear hierarchies include a text-based list of various virtual/multimedia situations (i.e., static, and active states of different types of feared animals) and 'in vivo'/live situations (e.g., petting a dog, touching a snake at a pet store). Therapy sessions 2-11 begin with a review of homework assigned during the prior week's session, followed by a ~35-minute virtual exposure session. Patients report their anxiety level using a 1-100 scale at a five-minute interval throughout the exposure and remain in the exposure until their self-reported anxiety reaches a 50% reduction from their peak anxiety rating from baseline following the introduction of the stimulus or 35 minutes elapses. That is, participants will spend a maximum of 35 minutes in the exposure and if they have reached a 50% reduction or greater from their peak anxiety rating before the 35 minutes is over, they will move on to the next exposure. Following the exposure, the therapist and patient process thoughts and feelings related to the exposure, reframe appraisals of their feared stimuli through cognitive restructuring, and practice box breathing. The Doxy.me VR group will conduct their in-session exposures in the VR clinic and the TMH group will conduct their in-session exposures using multimedia on the Doxy.me platform by screen sharing videos and photos. Once patients have progressed through all virtual situations in their hierarchy and are ready to complete their first in vivo exposure between therapy sessions for homework, weekly therapy sessions will be devoted to processing and planning between-session in vivo exposures.

Homework

All therapy sessions will include the assignment of homework, including reading an informational handout after the psychoeducation and treatment planning session (session 1) and exposure therapy assignments after therapy sessions 2-11. Daily exposure-based homework assignments will require participants to practice exposure exercises similar to those completed during that week's therapy session (i.e., using VR or multimedia stimuli) for a minimum of 30 minutes. When the patient states that they are ready, these will include in vivo exposure exercises planned during the therapy session. Participants will receive text/email reminders with links to REDCap surveys with instructions for the homework assignment and the situation to be used in the exposure exercise. The survey will also ask participants to report the date and time of the exposure, their baseline anxiety rating, their peak anxiety level during the exposure, and their anxiety level following completion of the exposure exercise.

5-x) Clarify the level of human involvement

Clarify the level of human involvement (care providers or health professionals, also technical assistance) in the e-intervention or as co-intervention (detail number and expertise of professionals involved, if any, as well as “type of assistance offered, the timing and frequency of the support, how it is initiated, and the medium by which the assistance is delivered”. It may be necessary to distinguish between the level of human involvement required for the trial, and the level of human involvement required for a routine application outside of a RCT setting (discuss under item 21 – generalizability).

	1	2	3	4	5	
subitem not at all important	○	○	○	○	◉	essential

Clear selection

Does your paper address subitem 5-x?

Copy and paste relevant sections from the manuscript (include quotes in quotation marks "like this" to indicate direct quotes from your manuscript), or elaborate on this item by providing additional information not in the ms, or briefly explain why the item is not applicable/relevant for your study

As stated above, all therapy sessions were completed with the study therapist. Study assessments and homework assignments were partially automated via REDCap.

5-xi) Report any prompts/reminders used

Report any prompts/reminders used: Clarify if there were prompts (letters, emails, phone calls, SMS) to use the application, what triggered them, frequency etc. It may be necessary to distinguish between the level of prompts/reminders required for the trial, and the level of prompts/reminders for a routine application outside of a RCT setting (discuss under item 21 – generalizability).

	1	2	3	4	5	
subitem not at all important	○	○	○	○	◉	essential

Clear selection

Does your paper address subitem 5-xi? *

Copy and paste relevant sections from the manuscript (include quotes in quotation marks "like this" to indicate direct quotes from your manuscript), or elaborate on this item by providing additional information not in the ms, or briefly explain why the item is not applicable/relevant for your study

This describes the partial automation and reminder system in REDCap: "All feasibility trial data will be collected via two REDCap projects. The first REDCap project will include a survey to collect responses to the Online Pre-Screening Questionnaire, which will include questions about basic eligibility criteria, the SMSP, and contact information. If potential participants meet initial study eligibility criteria the REDCap survey, they will be directed to a Microsoft Bookings page to schedule their baseline assessment, with automated reminders about the appointment 24 and 1 hour(s) prior to the appointment. The second, main REDCap project will facilitate all other data collection for the feasibility trial. Participants' Online Pre-Baseline Screening Questionnaire responses will be automatically transferred to the main clinical trial project using the REDCap piping function in preparation for the baseline assessment. After obtaining informed consent, supported by the REDCap e-Consent Framework feature, participants will be automatically directed to baseline questionnaire surveys, for which REDCap auto-calculate the scores. Following completion of the baseline assessment the participant will be randomized to their respective treatment conduction using the Randomization Module. All therapy session data (i.e., session number, date, time, length), adherence checklist, clinical notes, and in-session exposure ratings will be entered into a REDCap form by the study therapist during each therapy session. Following the completion of each session, participants will receive automated survey invitations to complete homework assignments, which will be delivered on specific days during the following based on the session number, session date, and treatment condition. Weekly post-session questionnaires (i.e., SIPQ, CSQ-VR) will be completed via REDCap surveys, displayed conditionally based on treatment condition (e.g., the CSQ-VR will only be displayed to those in the Doxy.me VR condition). Specific REDCap automations and features used for each stage of the trial are included in Figure 2. "

5-xii) Describe any co-interventions (incl. training/support)

Describe any co-interventions (incl. training/support): Clearly state any interventions that are provided in addition to the targeted eHealth intervention, as ehealth intervention may not be designed as stand-alone intervention. This includes training sessions and support [1]. It may be necessary to distinguish between the level of training required for the trial, and the level of training for a routine application outside of a RCT setting (discuss under item 21 – generalizability.

	1	2	3	4	5	
subitem not at all important	○	○	○	○	◉	essential

Clear selection

Does your paper address subitem 5-xii? *

Copy and paste relevant sections from the manuscript (include quotes in quotation marks "like this" to indicate direct quotes from your manuscript), or elaborate on this item by providing additional information not in the ms, or briefly explain why the item is not applicable/relevant for your study

"The present study will follow the treatment protocol developed specifically for VRET by Bouchard et al. [49–52] Following the baseline assessment session, treatment consists of 12, ≤ 60-minute therapy sessions, including one psychoeducation and treatment planning session (session 1), 10 exposure sessions (sessions 2-11), and one relapse prevention session (session 12). During the first therapy session, patients learn about the principles of Cognitive Behavioral Therapy (CBT), the etiology of anxiety and specific phobias, and procedures for exposure. They then complete a fear hierarchy (i.e., a list of situations ranked from least to most anxiety provoking) with therapist guidance. Fear hierarchies include a text-based list of various virtual/multimedia situations (i.e., static, and active states of different types of feared animals) and ‘in vivo’/live situations (e.g., petting a dog, touching a snake at a pet store). Therapy sessions 2-11 begin with a review of homework assigned during the prior week’s session, followed by a ~35-minute virtual exposure session. Patients report their anxiety level using a 1-100 scale at a five-minute interval throughout the exposure and remain in the exposure until their self-reported anxiety reaches a 50% reduction from their peak anxiety rating from baseline following the introduction of the stimulus or 35 minutes elapses. That is, participants will spend a maximum of 35 minutes in the exposure and if they have reached a 50% reduction or greater from their peak anxiety rating before the 35 minutes is over, they will move on to the next exposure. Following the exposure, the therapist and patient process thoughts and feelings related to the exposure, reframe appraisals of their feared stimuli through cognitive restructuring, and practice box breathing. The Doxy.me VR group will conduct their in-session exposures in the VR clinic and the TMH group will conduct their in-session exposures using multimedia on the Doxy.me platform by screen sharing videos and photos. Once patients have progressed through all virtual situations in their hierarchy and are ready to complete their first in vivo exposure between therapy sessions for homework, weekly therapy sessions will be devoted to processing and planning between-session in vivo exposures. "

6a) Completely defined pre-specified primary and secondary outcome measures, including how and when they were assessed

Does your paper address CONSORT subitem 6a? *

Copy and paste relevant sections from the manuscript (include quotes in quotation marks "like this" to indicate direct quotes from your manuscript), or elaborate on this item by providing additional information not in the ms, or briefly explain why the item is not applicable/relevant for your study

"We will assess feasibility of the proposed trial methodology using the following benchmarks: (1) 30 participants will be enrolled in months 1-9 of trial, (2) ≥70% of participants (n=21) will be retained for three-month follow-up assessments, (3) 70% of weekly self-report assessments will be completed, and (4) treatment fidelity we will be ≥80%. The small sample size of this feasibility trial will prevent us from making conclusions about efficacy; however, a Repeated Measures Analysis of Variance (rANOVA) will be used to conduct a preliminary assessment of between-group differences in clinical outcomes while co-varying for pre-treatment scores. We will also conduct a preliminary examination of associations between study targets (i.e., therapeutic alliance, presence) and homework adherence. Data obtained from these analyses will inform power analyses aimed at determining sample size requirements for a fully powered efficacy trial. "

6a-i) Online questionnaires: describe if they were validated for online use and apply CHERRIES items to describe how the questionnaires were designed/deployed

If outcomes were obtained through online questionnaires, describe if they were validated for online use and apply CHERRIES items to describe how the questionnaires were designed/deployed [9].

	1	2	3	4	5	
subitem not at all important	○	○	○	○	◉	essential

Clear selection

Does your paper address subitem 6a-i?

Copy and paste relevant sections from manuscript text

All assessments were chosen considering several factors including: (1) sound psychometric properties, (2) ease of administration, (3) past use in similar clinical trials, and (4) brevity. All assessments will be facilitated remotely by the study therapist and will occur at baseline, each session, mid-treatment (i.e., after completion of six therapy sessions, up to six-weeks post-baseline), and 12-weeks post-baseline. Self-report questionnaires will be completed by patients via REDCap survey with the study therapist present to answer any questions. Structured diagnostic interviews will be administered by study therapists and recorded in REDCap.

Specific Phobia Symptoms and Severity

The Diagnostic Assessment Research Tool (DART)[53]

[53] is a modular semi structured interview corresponding with the Diagnostic and Statistical Manual of Mental Disorders, 5th edition (DSM-5). [54] Study therapists will administer the DART Specific Phobia Module (DART-SP), which provides designations of Absent, Subthreshold, and Present. While the DART is a new tool, initial validation indicates excellent construct validity, discriminant validity, and convergent validity across modules. [53,55]

The Severity Measure for Specific Phobia for Adults (SMSP) [56] is a 10-item self-report questionnaire assessing the severity of specific phobia symptoms as they relate to the respondent's feared stimulus. Total scores range from 0 to 4 with higher scores indicating higher phobia severity. The SMSP has excellent internal consistency, criterion, and discriminant validity. [57]

Other Mental Health Symptoms

Suicide and Homicide Risk

The DART-Risk Assessment Module (DART-RA)[53] will be administered by study therapists to assess risk for suicidal and homicidal ideation, intention, and plans and guides the formation of safety plans where warranted.

Generalized Anxiety

The Generalized Anxiety Disorder-7 (GAD-7) [58] is a 7-item self-report measure of anxiety symptom severity. Scores range from 0 to 15 with higher scores indicating higher anxiety severity. The GAD-7 is widely used and has demonstrated excellent internal consistency reliability, good convergent validity.[59]

Depression

The Patient Health Questionnaire-8 (PHQ-8) [60] is an 8-item self-report measure of depression symptom severity. Scores range from 0 to 27 with higher scores indicating higher depression severity. The PHQ-8 has demonstrated excellent internal consistency [illegible]

reliability, good convergent validity, and specificity for depression diagnosis.[61]

Treatment-Related Factors

Working Alliance

The Working Alliance Inventory (WAI-SR) [62] is a 10-item client-rated measure of therapeutic alliance. Total scores range from 1 to 5 with higher scores indicating a better therapeutic alliance. The WAI-SR has demonstrated excellent internal consistency, reliability, and good convergent validity. [63]

Presence

The Single Item Presence Questionnaire (SIPQ)[64] is a 1-item self-reported measure of telepresence. Respondents are asked, "to what extent did you feel present in the environment, as if you were really there?" and provide ratings on a scale of 0 "not at all present" to 10 "'totally present." The SIPQ has demonstrated good to excellent content, face validity, test-retest, convergent and divergent validity, and sensitivity. [64]

Cybersickness

The Cybersickness in VR Questionnaire (CSQ-VR) [65]is an 8-item measure of nausea, vestibular, and oculomotor cybersickness experienced in VR. Scores range from 6 to 27 with higher scores indicating higher levels of cybersickness. The CSQ-VR has demonstrated excellent internal consistency and convergent validity. [66]

Client Satisfaction with Treatment

The Client Satisfaction Questionnaire-4 (CSQ-4) [67]is a 4-item client-rated measure satisfaction with treatment. Scores range from 0 to 16 with higher scores indicating higher satisfaction. The CSQ-4 has demonstrated good structural validity and internal reliability and is correlated with clinical outcomes and post-treatment functioning. [68]

System Usability

The System Usability Scale (SUS)[69] is a 10-item self-report measure of the usability of a particular software system, platform, or app. Scores range from 0-100 with higher scores indicating better usability. The SUS has demonstrated good internal consistency reliability and is a useful tool for comparing system usability between two or more platforms.

6a-ii) Describe whether and how “use” (including intensity of use/dosage) was defined/measured/monitored

Describe whether and how “use” (including intensity of use/dosage) was defined/measured/monitored (logins, logfile analysis, etc.). Use/adoption metrics are important process outcomes that should be reported in any ehealth trial.

	1	2	3	4	5	
subitem not at all important	○	○	○	○	◉	essential

Clear selection

Does your paper address subitem 6a-ii?

Copy and paste relevant sections from manuscript text

This was measured using therapy appointment attendance and homework completion rates.

6a-iii) Describe whether, how, and when qualitative feedback from participants was obtained

Describe whether, how, and when qualitative feedback from participants was obtained (e.g., through emails, feedback forms, interviews, focus groups).

	1	2	3	4	5	
subitem not at all important	◉	○	○	○	○	essential

Clear selection

Does your paper address subitem 6a-iii?

Copy and paste relevant sections from manuscript text

No qualitative feedback was obtained.

6b) Any changes to trial outcomes after the trial commenced, with reasons

Does your paper address CONSORT subitem 6b? *

Copy and paste relevant sections from the manuscript (include quotes in quotation marks "like this" to indicate direct quotes from your manuscript), or elaborate on this item by providing additional information not in the ms, or briefly explain why the item is not applicable/relevant for your study

There were no changes in trial outcomes.

7a) How sample size was determined

NPT: When applicable, details of whether and how the clustering by care provides or centers was addressed

7a-i) Describe whether and how expected attrition was taken into account when calculating the sample size

Describe whether and how expected attrition was taken into account when calculating the sample size.

	1	2	3	4	5	
subitem not at all important	(●)	( )	( )	( )	( )	essential

Clear selection

Does your paper address subitem 7a-i?

Copy and paste relevant sections from manuscript title (include quotes in quotation marks "like this" to indicate direct quotes from your manuscript), or elaborate on this item by providing additional information not in the ms, or briefly explain why the item is not applicable/relevant for your study

This was a feasibility trial and therefore recruitment was not based on power analysis.

7b) When applicable, explanation of any interim analyses and stopping guidelines

Does your paper address CONSORT subitem 7b? *

Copy and paste relevant sections from the manuscript (include quotes in quotation marks "like this" to indicate direct quotes from your manuscript), or elaborate on this item by providing additional information not in the ms, or briefly explain why the item is not applicable/relevant for your study

There were no interim analyses.

8a) Method used to generate the random allocation sequence

NPT: When applicable, how care providers were allocated to each trial group

Does your paper address CONSORT subitem 8a? *

Copy and paste relevant sections from the manuscript (include quotes in quotation marks "like this" to indicate direct quotes from your manuscript), or elaborate on this item by providing additional information not in the ms, or briefly explain why the item is not applicable/relevant for your study

We used the REDCap randomization module

8b) Type of randomisation; details of any restriction (such as blocking and block size)

Does your paper address CONSORT subitem 8b? *

Copy and paste relevant sections from the manuscript (include quotes in quotation marks "like this" to indicate direct quotes from your manuscript), or elaborate on this item by providing additional information not in the ms, or briefly explain why the item is not applicable/relevant for your study

As stated above, we used the REDCap randomization module with 1:1 allocation.

9) Mechanism used to implement the random allocation sequence (such as sequentially numbered containers), describing any steps taken to conceal the sequence until interventions were assigned

Does your paper address CONSORT subitem 9? *

Copy and paste relevant sections from the manuscript (include quotes in quotation marks "like this" to indicate direct quotes from your manuscript), or elaborate on this item by providing additional information not in the ms, or briefly explain why the item is not applicable/relevant for your study

The allocation sequence was generated in a spreadsheet and uploaded to REDCap randomization module, which conceals the allocation sequence throughout the clinical trial.

10) Who generated the random allocation sequence, who enrolled participants, and who assigned participants to interventions

Does your paper address CONSORT subitem 10? *

Copy and paste relevant sections from the manuscript (include quotes in quotation marks "like this" to indicate direct quotes from your manuscript), or elaborate on this item by providing additional information not in the ms, or briefly explain why the item is not applicable/relevant for your study

The study therapist generated the random allocation sequence, which was uploaded and concealed before the trial began. They also enrolled participants and used the REDCap randomization module, which automatically assigns participants to interventions.

11a) If done, who was blinded after assignment to interventions (for example, participants, care providers, those assessing outcomes) and how

NPT: Whether or not administering co-interventions were blinded to group assignment

11a-i) Specify who was blinded, and who wasn't

Specify who was blinded, and who wasn't. Usually, in web-based trials it is not possible to blind the participants [1, 3] (this should be clearly acknowledged), but it may be possible to blind outcome assessors, those doing data analysis or those administering co-interventions (if any).

	1	2	3	4	5	
subitem not at all important	(●)	( )	( )	( )	( )	essential

Clear selection

Does your paper address subitem 11a-i? *

Copy and paste relevant sections from the manuscript (include quotes in quotation marks "like this" to indicate direct quotes from your manuscript), or elaborate on this item by providing additional information not in the ms, or briefly explain why the item is not applicable/relevant for your study

No one was blinded after assignment to interventions.

11a-ii) Discuss e.g., whether participants knew which intervention was the "intervention of interest" and which one was the "comparator"

Informed consent procedures (4a-ii) can create biases and certain expectations - discuss e.g., whether participants knew which intervention was the "intervention of interest" and which one was the "comparator".

	1	2	3	4	5	
subitem not at all important	○	○	○	○	◉	essential

Clear selection

Does your paper address subitem 11a-ii?

Copy and paste relevant sections from the manuscript (include quotes in quotation marks "like this" to indicate direct quotes from your manuscript), or elaborate on this item by providing additional information not in the ms, or briefly explain why the item is not applicable/relevant for your study

Participants were informed of this during the consent.

11b) If relevant, description of the similarity of interventions

(this item is usually not relevant for ehealth trials as it refers to similarity of a placebo or sham intervention to a active medication/intervention)

Does your paper address CONSORT subitem 11b? *

Copy and paste relevant sections from the manuscript (include quotes in quotation marks "like this" to indicate direct quotes from your manuscript), or elaborate on this item by providing additional information not in the ms, or briefly explain why the item is not applicable/relevant for your study

As described above, the only difference between the intervention groups was the use of the VR clinic for exposures. Participants completed the same exposures for the same length of time.

12a) Statistical methods used to compare groups for primary and secondary outcomes

NPT: When applicable, details of whether and how the clustering by care providers or centers was addressed

Does your paper address CONSORT subitem 12a? *

Copy and paste relevant sections from the manuscript (include quotes in quotation marks "like this" to indicate direct quotes from your manuscript), or elaborate on this item by providing additional information not in the ms, or briefly explain why the item is not applicable/relevant for your study

"We will assess feasibility of the proposed trial methodology using the following benchmarks: (1) 30 participants will be enrolled in months 1-9 of trial, (2) ≥70% of participants (n=21) will be retained for three-month follow-up assessments, (3) 70% of weekly self-report assessments will be completed, and (4) treatment fidelity we will be ≥80%. The small sample size of this feasibility trial will prevent us from making conclusions about efficacy; however, a Repeated Measures Analysis of Variance (rANOVA) will be used to conduct a preliminary assessment of between-group differences in clinical outcomes while co-varying for pre-treatment scores. We will also conduct a preliminary examination of associations between study targets (i.e., therapeutic alliance, presence) and homework adherence. Data obtained from these analyses will inform power analyses aimed at determining sample size requirements for a fully powered efficacy trial. "

12a-i) Imputation techniques to deal with attrition / missing values

Imputation techniques to deal with attrition / missing values: Not all participants will use the intervention/comparator as intended and attrition is typically high in ehealth trials. Specify how participants who did not use the application or dropped out from the trial were treated in the statistical analysis (a complete case analysis is strongly discouraged, and simple imputation techniques such as LOCF may also be problematic [4]).

	1	2	3	4	5	
subitem not at all important	(●)	( )	( )	( )	( )	essential

Clear selection

Does your paper address subitem 12a-i? *

Copy and paste relevant sections from the manuscript (include quotes in quotation marks "like this" to indicate direct quotes from your manuscript), or elaborate on this item by providing additional information not in the ms, or briefly explain why the item is not applicable/relevant for your study

Data analysis is not yet completed. The strategy for handling missing data/attrition has not yet been determined.

12b) Methods for additional analyses, such as subgroup analyses and adjusted analyses

Does your paper address CONSORT subitem 12b? *

Copy and paste relevant sections from the manuscript (include quotes in quotation marks "like this" to indicate direct quotes from your manuscript), or elaborate on this item by providing additional information not in the ms, or briefly explain why the item is not applicable/relevant for your study

There are no additional planned analyses.

X26) REB/IRB Approval and Ethical Considerations [recommended as subheading under "Methods"] (not a CONSORT item)

X26-i) Comment on ethics committee approval

	1	2	3	4	5	
subitem not at all important	○	○	○	○	◉	essential

Clear selection

Does your paper address subitem X26-i?

Copy and paste relevant sections from the manuscript (include quotes in quotation marks "like this" to indicate direct quotes from your manuscript), or elaborate on this item by providing additional information not in the ms, or briefly explain why the item is not applicable/relevant for your study

"The trial was registered on ClinicalTrials.gov [NCT06302868] and the protocol was approved by the University of South Florida Institutional Review Board (#006215). "

x26-ii) Outline informed consent procedures

Outline informed consent procedures e.g., if consent was obtained offline or online (how? Checkbox, etc.?), and what information was provided (see 4a-ii). See [6] for some items to be included in informed consent documents.

	1	2	3	4	5	
subitem not at all important	○	○	○	○	◉	essential

Clear selection

Does your paper address subitem X26-ii?

Copy and paste relevant sections from the manuscript (include quotes in quotation marks "like this" to indicate direct quotes from your manuscript), or elaborate on this item by providing additional information not in the ms, or briefly explain why the item is not applicable/relevant for your study

"The consent and baseline assessment visit will be conducted via video-conferencing platform and will not be audio or video recorded. During this visit, study staff will (1) confirm the potential participant’s responses on the Online Pre-Screening Questionnaire; (2) provide detailed information about the study and obtain informed consent; (3) assist participants in completing baseline questionnaires via REDCap survey; (4) administer the Specific Phobia and Risk Assessment modules of the Diagnostic Assessment Research Tool (DART); and (5) make a final determination on the participant’s eligibility. Eligible participants will then be randomized using the REDCap Randomization Module and scheduled for their first treatment visit. "

X26-iii) Safety and security procedures

Safety and security procedures, incl. privacy considerations, and any steps taken to reduce the likelihood or detection of harm (e.g., education and training, availability of a hotline)

	1	2	3	4	5	
subitem not at all important	○	○	○	○	◉	essential

Clear selection

Does your paper address subitem X26-iii?

Copy and paste relevant sections from the manuscript (include quotes in quotation marks "like this" to indicate direct quotes from your manuscript), or elaborate on this item by providing additional information not in the ms, or briefly explain why the item is not applicable/relevant for your study

"Some questions asked during assessments and exposure exercises may cause participants distress, but this distress is expected to be similar to what participants would experience with routine care for specific fears/phobias. The study therapist will ensure that any questions causing distress are discussed with participants and provide them with techniques to reduce distress. A temporary increase in the severity of anxiety is expected at the beginning of exposure therapy, along with a gradual decline in severity over the course of treatment. If participants are distressed and unable to participate in the study following baseline assessments, or at the end of the study participants feel that they need further treatment, the study therapist will provide a referral to therapist(s) in the participants' local area. Referrals will be provided verbally to participants and the study therapist will follow up in one week via telephone to inquire as to the state of the referral and provide further assistance as necessary. If participants endorse suicidality or homicidality at any point during the study, we will follow the standard operational procedures of the University of South Florida Department of Psychiatry and Behavioral Neurosciences requiring safety planning with the participant at risk and mandatory reporting responsibilities. "

RESULTS

13a) For each group, the numbers of participants who were randomly assigned, received intended treatment, and were analysed for the primary outcome

NPT: The number of care providers or centers performing the intervention in each group and the number of patients treated by each care provider in each center

Does your paper address CONSORT subitem 13a? *

Copy and paste relevant sections from the manuscript (include quotes in quotation marks "like this" to indicate direct quotes from your manuscript), or elaborate on this item by providing additional information not in the ms, or briefly explain why the item is not applicable/relevant for your study

In total, 54 participants were enrolled, randomized, and treated between October 25th 2023 and July 26th 2024 (Doxy.me VR, n = 28, TMH, n= 26).

13b) For each group, losses and exclusions after randomisation, together with reasons

Does your paper address CONSORT subitem 13b? (NOTE: Preferably, this is shown in a CONSORT flow diagram) *

Copy and paste relevant sections from the manuscript (include quotes in quotation marks "like this" to indicate direct quotes from your manuscript), or elaborate on this item by providing additional information not in the ms, or briefly explain why the item is not applicable/relevant for your study

"The first participant was enrolled on October 25th, 2023, and the last therapy session and three-month post baseline assessment was completed on July 26th, 2024. In total, 54 participants were enrolled and randomized. See Figure 3 for the CONSORT diagram. Data analysis for the primary and secondary aims of the clinical trial will be completed and submitted by the end of the second quarter of 2025. "

13b-i) Attrition diagram

Strongly recommended: An attrition diagram (e.g., proportion of participants still logging in or using the intervention/comparator in each group plotted over time, similar to a survival curve) or other figures or tables demonstrating usage/dose/engagement.

	1	2	3	4	5	
subitem not at all important	◉	○	○	○	○	essential

Clear selection

Does your paper address subitem 13b-i?

Copy and paste relevant sections from the manuscript or cite the figure number if applicable (include quotes in quotation marks "like this" to indicate direct quotes from your manuscript), or elaborate on this item by providing additional information not in the ms, or briefly explain why the item is not applicable/relevant for your study

We did not include an attrition diagram though attrition is documented in the CONSORT diagram

14a) Dates defining the periods of recruitment and follow-up

Does your paper address CONSORT subitem 14a? *

Copy and paste relevant sections from the manuscript (include quotes in quotation marks "like this" to indicate direct quotes from your manuscript), or elaborate on this item by providing additional information not in the ms, or briefly explain why the item is not applicable/relevant for your study

The first participant was enrolled on October 25th, 2023, and the last therapy session and three-month post baseline assessment was completed on July 26th, 2024. In total, 54 participants were enrolled and randomized. See Figure 3 for the CONSORT diagram. Data analysis for the primary and secondary aims of the clinical trial will be completed and submitted by the end of the second quarter of 2025.

14a-i) Indicate if critical “secular events” fell into the study period

Indicate if critical “secular events” fell into the study period, e.g., significant changes in Internet resources available or “changes in computer hardware or Internet delivery resources”

	1	2	3	4	5	
subitem not at all important	◉	○	○	○	○	essential

Clear selection

Does your paper address subitem 14a-i?

Copy and paste relevant sections from the manuscript (include quotes in quotation marks "like this" to indicate direct quotes from your manuscript), or elaborate on this item by providing additional information not in the ms, or briefly explain why the item is not applicable/relevant for your study

There were no critical secular events during the study period

14b) Why the trial ended or was stopped (early)

Does your paper address CONSORT subitem 14b? *

Copy and paste relevant sections from the manuscript (include quotes in quotation marks "like this" to indicate direct quotes from your manuscript), or elaborate on this item by providing additional information not in the ms, or briefly explain why the item is not applicable/relevant for your study

The trial did not end early. It ended within the planned window.

15) A table showing baseline demographic and clinical characteristics for each group

NPT: When applicable, a description of care providers (case volume, qualification, expertise, etc.) and centers (volume) in each group

Does your paper address CONSORT subitem 15? *

Copy and paste relevant sections from the manuscript (include quotes in quotation marks "like this" to indicate direct quotes from your manuscript), or elaborate on this item by providing additional information not in the ms, or briefly explain why the item is not applicable/relevant for your study

We will report these results when we publish the clinical trial. This is just a protocol.

15-i) Report demographics associated with digital divide issues

In ehealth trials it is particularly important to report demographics associated with digital divide issues, such as age, education, gender, social-economic status, computer/Internet/ehealth literacy of the participants, if known.

	1	2	3	4	5	
subitem not at all important	(selected)	○	○	○	○	essential

Clear selection

Does your paper address subitem 15-i? *

Copy and paste relevant sections from the manuscript (include quotes in quotation marks "like this" to indicate direct quotes from your manuscript), or elaborate on this item by providing additional information not in the ms, or briefly explain why the item is not applicable/relevant for your study

We will report these results when we publish the clinical trial. This is just a protocol.

16) For each group, number of participants (denominator) included in each analysis and whether the analysis was by original assigned groups

16-i) Report multiple “denominators” and provide definitions

Report multiple “denominators” and provide definitions: Report N’s (and effect sizes) “across a range of study participation [and use] thresholds” [1], e.g., N exposed, N consented, N used more than x times, N used more than y weeks, N participants “used” the intervention/comparator at specific pre-defined time points of interest (in absolute and relative numbers per group). Always clearly define “use” of the intervention.

	1	2	3	4	5	
subitem not at all important	(●)	( )	( )	( )	( )	essential

Clear selection

Does your paper address subitem 16-i? *

Copy and paste relevant sections from the manuscript (include quotes in quotation marks "like this" to indicate direct quotes from your manuscript), or elaborate on this item by providing additional information not in the ms, or briefly explain why the item is not applicable/relevant for your study

We will report these results when we publish the clinical trial. This is just a protocol.

16-ii) Primary analysis should be intent-to-treat

Primary analysis should be intent-to-treat, secondary analyses could include comparing only “users”, with the appropriate caveats that this is no longer a randomized sample (see 18-i).

	1	2	3	4	5	
subitem not at all important	(●)	( )	( )	( )	( )	essential

Clear selection

Does your paper address subitem 16-ii?

Copy and paste relevant sections from the manuscript (include quotes in quotation marks "like this" to indicate direct quotes from your manuscript), or elaborate on this item by providing additional information not in the ms, or briefly explain why the item is not applicable/relevant for your study

We will report these results when we publish the clinical trial. This is just a protocol.

17a) For each primary and secondary outcome, results for each group, and the estimated effect size and its precision (such as 95% confidence interval)

Does your paper address CONSORT subitem 17a? *

Copy and paste relevant sections from the manuscript (include quotes in quotation marks "like this" to indicate direct quotes from your manuscript), or elaborate on this item by providing additional information not in the ms, or briefly explain why the item is not applicable/relevant for your study

We will report these results when we publish the clinical trial. This is just a protocol.

17a-i) Presentation of process outcomes such as metrics of use and intensity of use

In addition to primary/secondary (clinical) outcomes, the presentation of process outcomes such as metrics of use and intensity of use (dose, exposure) and their operational definitions is critical. This does not only refer to metrics of attrition (13-b) (often a binary variable), but also to more continuous exposure metrics such as “average session length”. These must be accompanied by a technical description how a metric like a “session” is defined (e.g., timeout after idle time) [1] (report under item 6a).

	1	2	3	4	5	
subitem not at all important	(●)	( )	( )	( )	( )	essential

Clear selection

Does your paper address subitem 17a-i?

Copy and paste relevant sections from the manuscript (include quotes in quotation marks "like this" to indicate direct quotes from your manuscript), or elaborate on this item by providing additional information not in the ms, or briefly explain why the item is not applicable/relevant for your study

We will report these results when we publish the clinical trial. This is just a protocol.

17b) For binary outcomes, presentation of both absolute and relative effect sizes is recommended

Does your paper address CONSORT subitem 17b? *

Copy and paste relevant sections from the manuscript (include quotes in quotation marks "like this" to indicate direct quotes from your manuscript), or elaborate on this item by providing additional information not in the ms, or briefly explain why the item is not applicable/relevant for your study

We will report these results when we publish the clinical trial. This is just a protocol.

18) Results of any other analyses performed, including subgroup analyses and adjusted analyses, distinguishing pre-specified from exploratory

Does your paper address CONSORT subitem 18? *

Copy and paste relevant sections from the manuscript (include quotes in quotation marks "like this" to indicate direct quotes from your manuscript), or elaborate on this item by providing additional information not in the ms, or briefly explain why the item is not applicable/relevant for your study

We will report these results when we publish the clinical trial. This is just a protocol.

18-i) Subgroup analysis of comparing only users

A subgroup analysis of comparing only users is not uncommon in ehealth trials, but if done, it must be stressed that this is a self-selected sample and no longer an unbiased sample from a randomized trial (see 16-iii).

	1	2	3	4	5	
subitem not at all important	(selected)	○	○	○	○	essential

Clear selection

Does your paper address subitem 18-i?

Copy and paste relevant sections from the manuscript (include quotes in quotation marks "like this" to indicate direct quotes from your manuscript), or elaborate on this item by providing additional information not in the ms, or briefly explain why the item is not applicable/relevant for your study

We will report these results when we publish the clinical trial. This is just a protocol.

19) All important harms or unintended effects in each group
(for specific guidance see CONSORT for harms)

Does your paper address CONSORT subitem 19? *

Copy and paste relevant sections from the manuscript (include quotes in quotation marks "like this" to indicate direct quotes from your manuscript), or elaborate on this item by providing additional information not in the ms, or briefly explain why the item is not applicable/relevant for your study

We will report these results when we publish the clinical trial. This is just a protocol.

19-i) Include privacy breaches, technical problems

Include privacy breaches, technical problems. This does not only include physical “harm” to participants, but also incidents such as perceived or real privacy breaches [1], technical problems, and other unexpected/unintended incidents. “Unintended effects” also includes unintended positive effects [2].

	1	2	3	4	5	
subitem not at all important	(•)	( )	( )	( )	( )	essential

Clear selection

Does your paper address subitem 19-i?

Copy and paste relevant sections from the manuscript (include quotes in quotation marks "like this" to indicate direct quotes from your manuscript), or elaborate on this item by providing additional information not in the ms, or briefly explain why the item is not applicable/relevant for your study

There were no privacy breaches or serious technical problems.

19-ii) Include qualitative feedback from participants or observations from staff/researchers

Include qualitative feedback from participants or observations from staff/researchers, if available, on strengths and shortcomings of the application, especially if they point to unintended/unexpected effects or uses. This includes (if available) reasons for why people did or did not use the application as intended by the developers.

	1	2	3	4	5	
subitem not at all important	(•)	( )	( )	( )	( )	essential

Clear selection

Does your paper address subitem 19-ii?

Copy and paste relevant sections from the manuscript (include quotes in quotation marks "like this" to indicate direct quotes from your manuscript), or elaborate on this item by providing additional information not in the ms, or briefly explain why the item is not applicable/relevant for your study

We did not collect qualitative feedback from participants.

DISCUSSION

22) Interpretation consistent with results, balancing benefits and harms, and considering other relevant evidence

NPT: In addition, take into account the choice of the comparator, lack of or partial blinding, and unequal expertise of care providers or centers in each group

22-i) Restate study questions and summarize the answers suggested by the data, starting with primary outcomes and process outcomes (use)

Restate study questions and summarize the answers suggested by the data, starting with primary outcomes and process outcomes (use).

	1	2	3	4	5	
subitem not at all important	○	○	◉	○	○	essential

Clear selection

Does your paper address subitem 22-i? *

Copy and paste relevant sections from the manuscript (include quotes in quotation marks "like this" to indicate direct quotes from your manuscript), or elaborate on this item by providing additional information not in the ms, or briefly explain why the item is not applicable/relevant for your study

"There will be a growing need for innovative solutions to enhance the effectiveness of remote care as TMH services continue to evolve. [1,16,17] VR has tremendous potential to increase the efficacy of evidence-based practices that were originally designed for in-person treatment but are now delivered via TMH with increased regularity (e.g., exposure therapy). This feasibility trial comparing exposure therapy via Doxy.me VR versus TMH for adults with intense fear of dogs, snakes, and/or spiders fills an important gap in the nascent research on telemedicine-based VR exposure therapy. Using this protocol, we will assess the feasibility of our proposed study methodology in preparation for a fully powered efficacy trial. We anticipate meeting or exceeding these feasibility criteria, which will provide valuable insights into the feasibility of conducting a large-scale efficacy trial.

Principal Findings

As anticipated, we exceeded our recruitment goal of enrolling 30 or more participants in months 1-9 of the clinical trial by enrolling 54 participants during months 1 –7. Data analysis will be completed and submitted by the second quarter of 2025 at which point we will report on the remaining feasibility results.

Comparison with Prior Work

Our study builds upon previous research demonstrating the efficacy of VR exposure to enhance treatment delivery. While there is significant support for in-clinic VR mental health therapy, evidence for telemedicine-based VR mental health is limited. Our study addresses this important gap by evaluating the feasibility and preliminary efficacy of exposure therapy via an innovative telemedicine-based VR clinic (i.e., Doxy.me VR), thereby contributing to the nascent research on the use of VR in telemental health care.

Limitations

The small sample size proposed by the current study limits the generalizability of our findings and prevents us from drawing conclusions about treatment efficacy. A large-scale efficacy trial will address this limitation.

Conclusions

This feasibility RCT comparing Doxy.me VR vs. TMH represents an important step towards leveraging VR technology to enhance the delivery of evidence-based treatments via telemedicine. By evaluating the feasibility and preliminary efficacy of exposure therapy via a telemedicine-based VR clinic, we will contribute a potential solution to common barriers to remotely delivered exposure therapy.

Future Directions

Future research will involve fully powered efficacy trials on telemedicine-based VR and the potential of this technology to improve treatment outcomes for mental health disorders."

22-ii) Highlight unanswered new questions, suggest future research

Highlight unanswered new questions, suggest future research.

	1	2	3	4	5	
subitem not at all important	◉	○	○	○	○	essential

Clear selection

Does your paper address subitem 22-ii?

Copy and paste relevant sections from the manuscript (include quotes in quotation marks "like this" to indicate direct quotes from your manuscript), or elaborate on this item by providing additional information not in the ms, or briefly explain why the item is not applicable/relevant for your study

This is a protocol paper.

20) Trial limitations, addressing sources of potential bias, imprecision, and, if relevant, multiplicity of analyses

20-i) Typical limitations in ehealth trials

Typical limitations in ehealth trials: Participants in ehealth trials are rarely blinded. Ehealth trials often look at a multiplicity of outcomes, increasing risk for a Type I error. Discuss biases due to non-use of the intervention/usability issues, biases through informed consent procedures, unexpected events.

	1	2	3	4	5	
subitem not at all important	○	○	◉	○	○	essential

Clear selection

Does your paper address subitem 20-i? *

Copy and paste relevant sections from the manuscript (include quotes in quotation marks "like this" to indicate direct quotes from your manuscript), or elaborate on this item by providing additional information not in the ms, or briefly explain why the item is not applicable/relevant for your study

"The small sample size proposed by the current study limits the generalizability of our findings and prevents us from drawing conclusions about treatment efficacy. A large-scale efficacy trial will address this limitation. "

21) Generalisability (external validity, applicability) of the trial findings

NPT: External validity of the trial findings according to the intervention, comparators, patients, and care providers or centers involved in the trial

21-i) Generalizability to other populations

Generalizability to other populations: In particular, discuss generalizability to a general Internet population, outside of a RCT setting, and general patient population, including applicability of the study results for other organizations

	1	2	3	4	5	
subitem not at all important	○	○	○	○	◉	essential

Clear selection

Does your paper address subitem 21-i?

Copy and paste relevant sections from the manuscript (include quotes in quotation marks "like this" to indicate direct quotes from your manuscript), or elaborate on this item by providing additional information not in the ms, or briefly explain why the item is not applicable/relevant for your study

"The small sample size proposed by the current study limits the generalizability of our findings and prevents us from drawing conclusions about treatment efficacy. A large-scale efficacy trial will address this limitation. "

21-ii) Discuss if there were elements in the RCT that would be different in a routine application setting

Discuss if there were elements in the RCT that would be different in a routine application setting (e.g., prompts/reminders, more human involvement, training sessions or other co-interventions) and what impact the omission of these elements could have on use, adoption, or outcomes if the intervention is applied outside of a RCT setting.

	1	2	3	4	5	
subitem not at all important	○	○	◉	○	○	essential

Clear selection

Does your paper address subitem 21-ii?

Copy and paste relevant sections from the manuscript (include quotes in quotation marks "like this" to indicate direct quotes from your manuscript), or elaborate on this item by providing additional information not in the ms, or briefly explain why the item is not applicable/relevant for your study

The clinical trial was conducted is a very similar manner to typical tele-health therapy sessions with exception of randomization.

OTHER INFORMATION

23) Registration number and name of trial registry

Does your paper address CONSORT subitem 23? *

Copy and paste relevant sections from the manuscript (include quotes in quotation marks "like this" to indicate direct quotes from your manuscript), or elaborate on this item by providing additional information not in the ms, or briefly explain why the item is not applicable/relevant for your study

The trial was registered on ClinicalTrials.gov [NCT06302868]

24) Where the full trial protocol can be accessed, if available

Does your paper address CONSORT subitem 24? *

Cite a Multimedia Appendix, other reference, or copy and paste relevant sections from the manuscript (include quotes in quotation marks "like this" to indicate direct quotes from your manuscript), or elaborate on this item by providing additional information not in the ms, or briefly explain why the item is not applicable/relevant for your study

The trial was registered on ClinicalTrials.gov [NCT06302868]

25) Sources of funding and other support (such as supply of drugs), role of funders

Does your paper address CONSORT subitem 25? *

Copy and paste relevant sections from the manuscript (include quotes in quotation marks "like this" to indicate direct quotes from your manuscript), or elaborate on this item by providing additional information not in the ms, or briefly explain why the item is not applicable/relevant for your study

Research reported in this publication was supported by the National Institute of Mental Health of the National Institutes of Health under Award Number R43MH129065. The content is solely the responsibility of the authors and does not necessarily represent the official views of the National Institutes of Health.

X27) Conflicts of Interest (not a CONSORT item)

X27-i) State the relation of the study team towards the system being evaluated

In addition to the usual declaration of interests (financial or otherwise), also state the relation of the study team towards the system being evaluated, i.e., state if the authors/evaluators are distinct from or identical with the developers/sponsors of the intervention.

	1	2	3	4	5	
subitem not at all important	○	○	○	○	◉	essential

Clear selection

Does your paper address subitem X27-i?

Copy and paste relevant sections from the manuscript (include quotes in quotation marks "like this" to indicate direct quotes from your manuscript), or elaborate on this item by providing additional information not in the ms, or briefly explain why the item is not applicable/relevant for your study

Drs. Bunnell, Ong, and Welch are employees of Doxy.me Inc., a commercial telemedicine company. The authors declare no other conflicts of interest.

About the CONSORT EHEALTH checklist

As a result of using this checklist, did you make changes in your manuscript? *

- ○ yes, major changes
- ◉ yes, minor changes
- ○ no

What were the most important changes you made as a result of using this checklist?

I added some key information to our procedures section.

How much time did you spend on going through the checklist INCLUDING making changes in your manuscript *

I spent around 1.5 hours to two hours

As a result of using this checklist, do you think your manuscript has improved? *

- (x) yes
- ( ) no
- ( ) Other:

Would you like to become involved in the CONSORT EHEALTH group?

This would involve for example becoming involved in participating in a workshop and writing an "Explanation and Elaboration" document

- ( ) yes
- (x) no
- ( ) Other:

Clear selection

Any other comments or questions on CONSORT EHEALTH

Your answer

STOP - Save this form as PDF before you click submit

To generate a record that you filled in this form, we recommend to generate a PDF of this page (on a Mac, simply select "print" and then select "print as PDF") before you submit it.

When you submit your (revised) paper to JMIR, please upload the PDF as supplementary file.

Don't worry if some text in the textboxes is cut off, as we still have the complete information in our database. Thank you!

Final step: Click submit !

Click submit so we have your answers in our database!

Submit Clear form

Never submit passwords through Google Forms.

This content is neither created nor endorsed by Google. - Terms of Service - Privacy Policy

Does this form look suspicious? Report

Google Forms
